# The cAMP effectors, Rap2b and EPAC, are involved in the regulation of the development of the *Coxiella burnetii* containing vacuole by altering the fusogenic capacity of the vacuole

**DOI:** 10.1371/journal.pone.0212202

**Published:** 2019-02-14

**Authors:** María Eugenia Mansilla Pareja, Maria Celeste Gaurón, Esteban Robledo, Milton Osmar Aguilera, María Isabel Colombo

**Affiliations:** 1 Laboratorio de Biología Celular y Molecular- Instituto de Histología y Embriología (IHEM)-Universidad Nacional de Cuyo, CONICET, Mendoza, Argentina; 2 Facultad de Ciencias Médicas, Mendoza, Argentina; 3 Facultad de Farmacia y Bioquímica, Universidad Juan Agustín Maza, Mendoza, Argentina; 4 Facultad de Odontología, UNCuyo, Mendoza, Argentina; Purdue University, UNITED STATES

## Abstract

Cyclic Adenosine 3′,5′-monophosphate (cAMP) is a key second messenger known to directly regulate not only the protein kinase A (PKA) activity but also other important molecules such as the exchange protein activated by cAMP (EPAC), which is as a guanine nucleotide exchange factor (GEF) of the low molecular weight GTPase, Rap2. *Coxiella burnetii* is a Gram negative bacterium that survives and grows in a large *Coxiella* replicative vacuole (CRV), which displays lysosomal and autophagic features. In this report, we present evidence that both, EPAC and its downstream effector Rap2b, were recruited to the CRV. The transient over-expression of the Rap2b wt protein, but not its inactive mutant Rap2b ΔAAX, markedly inhibited the development of the large CRV. Additionally, Rap2b wtinhibited the fusion of early *Coxiella* phagosomes with the fully developed CRV, indicating that homotypic fusion events are altered in the presence of high levels of Rap2b wt. Likewise, the fusion of endosome/lysosomal compartments (heterotypic fusions) with the large CRV was also affected by the over-expression of this GTPase. Interestingly, cell overexpression of Rap2b wt markedly decreased the levels of the v-SNARE, Vamp7, suggesting that this down-regulation impairs the homotypic and heterotypic fusions events of the *Coxiella* vacuole.

## Introduction

*Coxiella burnetii*, the etiological agent of the Q-fever, is an obligate intracellular bacterium. This microorganism has a biphasic life cycle in which the small cell variant (SCV) and the large cell variant (LCV) alternate. The LCV form is generated in the lysosomal-like large replicative compartment (*C*. *burnetii*-replicative vacuoles, CRVs) of infected cells, where the acidic microenvironment favors its replication [1]. In previous works, we have demonstrated that *C*. *burnetii* invades and travels within the host cell through classical endocytic/phagocytic compartments [2]. Furthermore, we have proved that *C*. *burnetii* persists and replicates in a large replicative vacuole, which displays autophagic features, and have hypothesized that certain components of the autophagic machinery favors the expansion of the CRV at early times post-infection (p.i.) [3] [4]. In order to modulate the recruitment of critical cellular proteins, *C*. *burnetii* releases numerous secretion proteins into the host cell by its type IV secretion system (T4SS) [5] [6]. Therefore, *C*. *burnetii* has the capacity of inducing the fusion of its CRV with several intracellular compartments, thus promoting the generation and growth of the vacuole. This process requires constant bacterial protein synthesis [7]. We have demonstrated that vesicles derived from the early secretory pathway also contribute to the growth of the large CRV probably by providing membranous components [8]. Furthermore, published works from our group and colleagues have demonstrated the contribution of SNAREs proteins (Vamp8, Vamp7, Vamp3, and Syntaxin 17) in homotypic and heterotypic fusion events that contribute to develop the replicative vacuole [7] [9].

The autophagy pathway is a highly conserved, physiological degradation process in eukaryotic cells. During autophagy, small portions of cytoplasm or damaged organelles are sequestered into double-membrane vesicles named autophagosomes. These vesicles then fuse with degradatives organelles which supply the hydrolytic enzymes for breaking down and eventual recycling of the sequestered material. The Microtubule-associated protein light chain 3 (LC3) has been shown to be an autophagosomal marker in mammals. There are two forms of LC3 called LC3-I and LC3-II, which are produced post-translationally in various cell types. LC3-I is a cytosolic protein, whereas LC3-II is membrane-bound and specifically associates with autophagosome membranes.

The autophagy pathway is activated in response to many physiological situations, acting as either a homeostasis control mechanism to eliminate unnecessary structures or as an adaptive response to adverse conditions, such as nutrients deprivation or starvation [10] [11] [12]. Many pathological stress conditions, such as pathogen invasion can also trigger autophagy since this process is a critical cell defense mechanism. Nevertheless, numerous intracellular pathogens, including *Staphylococcus aureus* and *Legionella pneumophila*, exploit the autophagy pathway for their own benefit in order to survive and replicate in the host cells. [13].

Cyclic AMP is a second messenger that controls numerous biological processes [14]. Many pathogens have developed mechanisms to increase intracellular cAMP levels to facilitate their survival within the phagosome. Interestingly, increased cAMP levels inhibit the acidification of phagosomes and phagosome-lysosome fusions, preventing phagosomal maturation [15]. It is known that the classical cAMP effector is PKA, which presents a cAMP-binding domain that has been evolutionary conserved. Interestingly, there is evidence indicating that PKA activation by cAMP leads to the inhibition of the autophagic pathway through a mechanism that involves the phosphorylation of LC3 [16], suggesting that cAMP is a modulator of the autophagy pathway.

In addition, it is known that cAMP directly regulates EPAC, which is a GEF of Rap GTPases, proteins involved in many cellular processes. [17] [18]. The Rap family comprises five members: two isoforms of Rap1 (Rap1a and Rap1b) and three isoforms of Rap2 (Rap2a, Rap2b and Rap2c), that share 70% homology with Rap1. An important difference between these two subfamilies is that Rap2 is less sensitive to its GTPase activating protein (GAP), which allows a signaling cascade that is sustained over time.

Rap activation is regulated by specific GEFs, such as CD-GEF1, C3G, PDZ-GEF1, and the EPACs [19] [20]. The activation of Rap through EPAC has been shown to regulate phagocytosis and vesicle trafficking. It has been demonstrated that Rap1 associates with the late endocytic compartment, while Rap2 associates with GTPases involved in Golgi-ER vesicle transport [21]. Besides, EPAC and Rap2b mediate the response induced by elevated intracellular cAMP levels, inhibiting the autophagy pathway [22]. We have previously demonstrated that the activation of the cAMP pathway, through EPAC and Rap proteins, leads to the inhibition of the autophagic host response induced by infection with *S*. *aureus* [23]. That study provided the first evidence indicating that the proteins EPAC and Rap2b are recruited as signaling molecules to a fraction of phagosomes containing *S*. *aureus* [23].

In this work, we aimed at studying the effectors EPAC and Rap2b as key regulators of CRV development. We have demonstrated that the cAMP modulated protein EPAC was recruited to the CRV. In addition by analyzing the EPAC downstream effector Rap2b, we determined that the latter factor, but not its inactive mutant Rap2b ΔAAX, is also recruited to the CRV from early times p.i. More importantly, we demonstrated that over-expression of Rap2b wt protein, but not Rap2b ΔAAX, significantly impaired the development of the large CRV. Interestingly, we have shown that the over-expression of Rap2b wt reduced both, the homotypic and the heterotypic fusion capacity of the CRV, and also, decreased the intracellular levels of the v-SNARE Vamp7. These results suggest that the over-expression of the active form of Rap2b affects molecular components of the fusion machinery that *C*. *burnetii* co-opt to generate its replicative vacuole. The results obtained in this work offer a deeper insight into the molecular components of the host cell that are involved in the regulatory mechanism of the development of *C*. *burnetii* replicative vacuole.

## Materials and methods

### Materials

D-MEM and alpha-MEM were obtained from Gibco Laboratories (Invitrogen, Argentina); fetal bovine serum (FBS) was obtained from GIBCO BRL/Life Technologies (Buenos Aires, Argentina). The anti-Rap2b antibody and Rap2b siRNA were purchased from Santa Cruz Biotechnology (Buenos Aires, Argentina). Rabbit anti-*Coxiella* antiserum and mCherry-*Coxiella burnetii* were generously provided by Dr. Robert Heinzen (Rocky Mountain Laboratories, NIAID, NIH, Hamilton, MT, USA). *Coxiella*-GFP (Tn1832) and the DotA mutant GFP (Tn292) were kindly provided by Dr. Matteo Bonazzi (Cell Biology of Bacterial Infections, UMR 5236 CPBS, Montpellier, France). Texas Red-tagged dextran (3 kDa) was purchased from Molecular Probes. PEGFP-Rap2b wt and pEGFP-Rap2b ΔAAX plasmids were kindly provided by Dr. MauroTorti (University of Pavia, Pavia, Italy). Plasmids for EPAC-GFP, Δ(1–148)-EPAC-GFP, Δ(72–148)-EPAC-GFP were kindly provided by Dr. Xiaodong Cheng (The University of Texas Medical Branch, Galveston, Texas).

### Cell culture

Vero cells (ATCC, CCL-81), HeLa cells (ATCC, CCL-2) and Chinese hamster ovary cells (CHO) (ATCC, CCL-61) were grown in 24-well plates in either D-MEM or α-MEM, supplemented with 15% FBS, and the antibiotics streptomycin (50μg/ml) and penicillin (50 μg/ml), at 37ºC in a 5% CO_2_ atmosphere until 80% confluence was reached.

### Cell transfection

CHO cells were transfected with the plasmids (1 μg/μl) using the Lipofectamine 2000 reagent (Invitrogen, Argentina) as previously described in [7]. Transfected cells were incubated for 24h in DMEM or α-MEM supplemented with 10% FBS.

### Propagation of phase II *C*. *burnetii*

The generation of *C*. *burnetii* clone 4, phase II, Nine Mile strain was performed as previously described [4].

### Infection of cells with *C*. *burnetii*

A volume of 0.5–1 ml of a dilution of *C*. *burnetii* suspension was added to cells plated on coverslips distributed in either 6 or 24 well plates. Afterwards, infected cells were incubated at 37°C in a 5% CO2 atmosphere for the indicated time periods [7]. In all experiments host cells were infected at a MOI (multiplicity of infection) of 10.

### Confocal microscopy

Transfected and infected CHO cells were analyzed by confocal microscopy using an Olympus FluoViewTM FV1000 confocal microscope (Olympus, Argentina), with the FV10-ASW (version 01.07.00.16) software. Images were processed using Adobe CS3 (Adobe Systems) and ImageJ. Confocal images (0.39-μm sections) were collected.

### Indirect immunofluorescence

Cells grown on coverslips were fixed with 4% paraformaldehyde for 15 min at room temperature, washed with PBS, and incubated with a quenching solution (50 mM NH4Cl in PBS). Cells were then permeabilized with a solution containing 1% saponin in PBS and 1% BSA. After permeabilization cells were incubated with the primary antibody diluted in PBS. Cells were then washed and incubated with a conjugated secondary antibody (Jackson immune Research Laboratories, EE.UU). After washing with PBS, cells were mounted with Mowiol (plus Hoechst solution) and examined by confocal microscopy as described previously [7].

### Measurement of the percentage of infected cells and the number and size of CRV

For fixation cell were incubated in 4% paraformaldehyde for 10 min. About 200 cells per coverslip (in triplicate) were scored for the presence or absence of large *C*. *burnetii* vacuoles using a confocal microscope (Olympus FV1000) with a 60x objective. Infected cells were defined as those with at least one large CRV (size ≥ 2μm) with clear identifiable bacteria inside. The vacuole size was determined by a morphometric analysis using the FV10-ASW or ImageJ software.

### Bacterial viability and replication

A fluorescent infectious FFU assay was used to quantify the replication and viability of *C*. *burnetii* in CHO cells. Infected cells were lysed with sterile distilled water and samples were serially diluted. Vero cells were then infected with these lysates in a 24 well plate as previously described [7], [24], [25]. After 72 h of infection, cells were fixed and processed for fluorescence microscopy. Approximately 1,000 cells were scored per coverslip.

### SDS-PAGE and western blot

Transfected and infected CHO cells were lysed with sample buffer. Protein samples were run on either a 12% or 15% polyacrylamide gels and transferred to Hybond-ECL (Amersham) nitrocellulose membranes. Membranes were blocked for 1 h in 5% non-fat milk, 0.05% Tween 20, and PBS at room temperature, washed twice with PBS and incubated with an anti-LC3 antibody followed by a peroxidase-conjugated secondary antibody (Jackson Immuno Research). An anti-tubulin antibody (Jackson Immuno Research) was used as a loading control. The corresponding bands were detected using an enhanced chemiluminescence (ECL) detection kit from (GE Healthcare, Amersham, RPN2109) using a Fujifilm LAS-4000 and analysis in ImageJ (NIH) software.

### siRNA silencing of EPAC or Rap2b

Purified siRNA against human EPAC or Rap2b or a control siRNA were purchased from BIONEER (Korea). Confluent HeLa cells were transfected with EPAC or Rap2b siRNA or with a control siRNA, all prepared at a final concentration of 20 nM in 400 ml of D-MEM without serum and with the Lipofectamine 2000 reagent. The mixture was added to a 24-well culture dish. After 5 h, the transfection mixture was replaced by fresh D-MEM with 10% FBS. At 48 h post-transfection the medium was removed and the same transfection protocol with the corresponding siRNA was applied again (second hit). When the second transfection mixture was removed, cells were infected with *C*. *burnetii* for 48 h at 37°C.

### Homotypic fusion assays

CHO cells were infected with *C*. *burnetii* (MOI = 10) and after 24h of infection cells were transfected with wt pEGFP-Rap2b or pEGFP- Rap2bΔAAX. At 48h of infection, cells were allowed to internalize mCherry-*Coxiella*. The 24 wells plate was centrifuged for 10 minutes (1,500 rpm) at 4°C in order to induce the contact of the mCherry-*Coxiella* (red) to the cell surface. After 2h of incubation cells were fixed and subjected to indirect immunofluorescence using a polyclonal antibody against *C*. *burnetii*. The glass slides were mounted and analyzed by confocal microscopy [7].

### Heterotypic fusion assays

HeLa or CHO cells were incubated with 5 μg/ml dextran-rhodamine for 2h and then transfected with pEGFP-Rap2b wt or pEGFP- Rap2bΔAAX. After 24 h of transfection, cells were infected with *C*. *burnetii* (MOI = 10). The 24 wells plate was centrifuged for 10 minutes (1,500 rpm) at 4°C in order to induce contact of the bacteria with the cell surface. After 2h of incubation, cells were washed three times with PBS, fixed and subjected to indirect immunofluorescence using a polyclonal antibody against *C*. *burnetii*. The glass slides were mounted and analyzed by confocal microscopy [7].

## Results

### EPAC is recruited to the *C*. *burnetii* replicative vacuole

It is known that Rap2b is involved in the regulation of the autophagic response induced by the toxin Hla from *S*. *aureus* [23]. We have demonstrated that the direct activation of EPAC and/or Rap2b inhibits the autophagic response induced by the toxin. Therefore, we determined if this was a common mechanism involved in the regulation of other intracellular pathogens, thus, we wondered whether these molecules also play a role in development of the CRV in *C*. *burnetii* infected cells[26].

**Fig 1:**
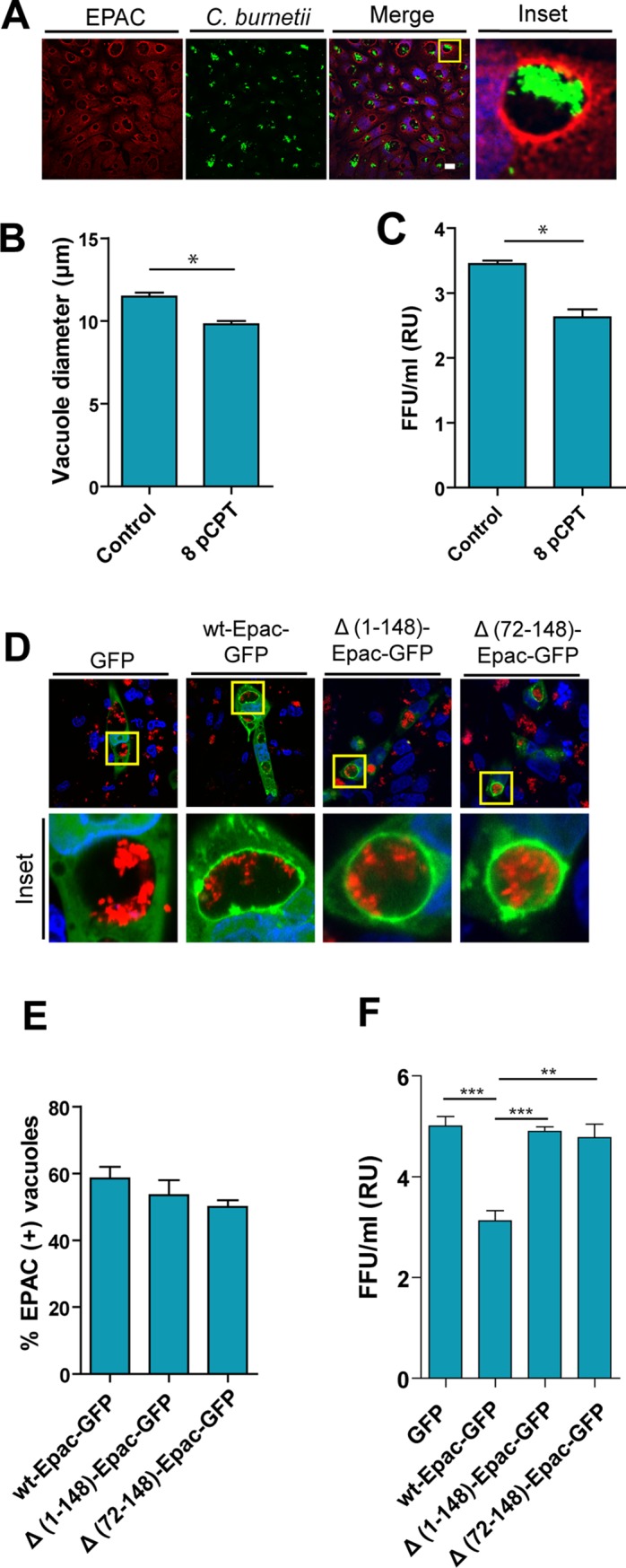
The presence of 8pCPT alters the number and the diameter of CRVs. **A.** CHO cells were infected with *C*. *burnetii* for 48 h (MOI = 10), fixed and subjected to indirect immunofluorescence using antibodies to detect endogenous EPAC protein (red) and against *C*. *burnetii* to detect the bacteria (green). The cells were analyzed by confocal microscopy. **B.** Quantification of the diameter of the vacuole in complete medium in the absence (Control) or the presence of 8-pCPT-cAMP (8pCPT). Data represent the mean± SEM of at least three independent experiments in which no less than 200 vacuoles were scored in each experiment. **C.** CHO cells were incubated in the complete medium in the absence (Control) or presence of 8-pCPT-cAMP (8pCPT) and infected for 48 h with *C*. *burnetii* to allow the development of the *Coxiella* vacuole. The cells were then lysed by sonication and the supernatant was diluted (1:100) and used to infect Vero cells. After 72 h of incubation (chase), cells were fixed and examined by fluorescence microscopy. Data represent the mean ± SEM of at least three independent experiments where a minimum of 1,000 cells were scored in each experiment (*p ≤0.05.). **D.** CHO cells were transfected with wt-EPAC-GFP, Δ(1–148)-EPAC-GFP, Δ(72–148)-EPAC-GFP and infected with *C*. *burnetii*. At 48 h, the cells were fixed and analyzed by confocal microscopy. **E.** Quantification of the percentage of wt-EPAC-GFP, Δ(1–148)-EPAC-GFP, Δ(72–148)-EPAC-GFP positive vacuoles. **F.**CHO cells were infected for 48 h with *C*. *burnetii* to allow the development of the *Coxiella* vacuole. The cells were then lysed by sonication and the supernatant was diluted (1:100) and used to infect Vero cells. After 72 h of incubation (chase), cells were fixed and examined by fluorescence microscopy. The data represent the mean ± SEM of at least 3 independent experiments. Tukey test * p ≤ 0.05. Bars = 10 μm.

To assess the presence of EPAC in the CRV membrane, CHO cells were infected with *C*. *burnetii* for 48 h and fixed. The presence of EPAC was then evaluated by indirect immunofluorescence employing anti-EPAC antibody (Fig 1A). A marked recruitment of EPAC to the *Coxiella*-containing phagosomes was observed (Fig 1A). In order to evaluate the participation of EPAC in the maturation and growth of the vacuole, 8-pCPT-2'-O Me-cAMP (8-pCPT-cAMP), a cAMP analog that preferentially activates EPAC [18] was employed. To that end, CHO cells were incubated in either the absence or the presence of 8-pCPT-cAMP and infected with *C*. *burnetii* for 48 h. A moderate but statistically significant decrease in the vacuole diameter of cells treated with this compound was observed (Fig 1B). This result suggests that the direct and specific stimulation of EPAC inhibits the expansion of the vacuole. Therefore, to study the role of 8-pCPT-cAMP in bacterial replication, a focus-forming unit (FFU) assay was performed in CHO cells infected with *C*. *burnetii* for 48h and incubated in either complete medium (control) or in complete medium containing 8-pCPT-cAMP (8pCPT). As shown in Panel C in the presence of 8pCPT-cAMP there was a decrease in the *Coxiella* replication suggesting that EPAC activation inhibits bacterial growth.

To determine the requirements for EPAC association to the CRV membrane, CHO cells were transfected with wt-EPAC-GFP and the truncated forms Δ(1–148)-EPAC-GFP and Δ(72–148)-EPAC-GFP, and then infected with *C*. *burnetii*. At 48 h p.i., cells were fixed and analyzed by Laser Scanning Confocal Microscopy (LSCM). The Δ(1–148)-EPAC-GFP deletion mutant lacks the first 148 amino acid residues that are required for EPAC targeting, whereas, the Δ(72–148) EPAC-GFP lacks the DEP domain that alters the EPAC nuclear membrane binding motif. [27]. This mutant allowed demonstrating the role of the DEP domain in membrane targeting of EPAC but not its mitochondrial association [27]. Fig 1D shows the recruitment of wt-EPAC-GFP, Δ(1–148)-EPAC-GFP and Δ(72–148)-EPAC-GFP on the CRV membrane. As depicted in Fig 1E 50–60% of the *Coxiella*-containing phagosomal compartments were labeled by wt-EPAC-GFP and the corresponding truncated forms. These results suggest that neither, the N-terminal sequence nor the DEP domain of EPAC is involved in the recruitment mechanism of EPAC to the *C*. *burnetti* vacuole. Thus, EPAC might be recruited to the CRV membrane by a different mechanism likely involving bacterial proteins.

To assess the role of EPAC in bacterial replication, a FFU assay was performed in CHO cells transiently overexpressing either wt-EPAC-GFP or the inactive mutants Δ(1–148)-EPAC-GFP and Δ(72–148)-EPAC-GFP plasmids, and infected with *C*. *burnetii* for 48h. Significant differences in the replicative capacity of *C*. *burnetii* were observed in wt-EPAC-GFP transfected cells in comparison with GFP control and the inactive truncated mutants Δ(1–148)-EPAC-GFP Δ(72–148)-EPAC-GFP (Fig 1F). This result is in agreement with the evidence that the presence of 8pCPT-cAMP produced a decrease in the *Coxiella* replication as shown above.

### Rap2b is recruited to the *C*. *burnetii* replicative vacuole from early post infection times

**Fig 2:**
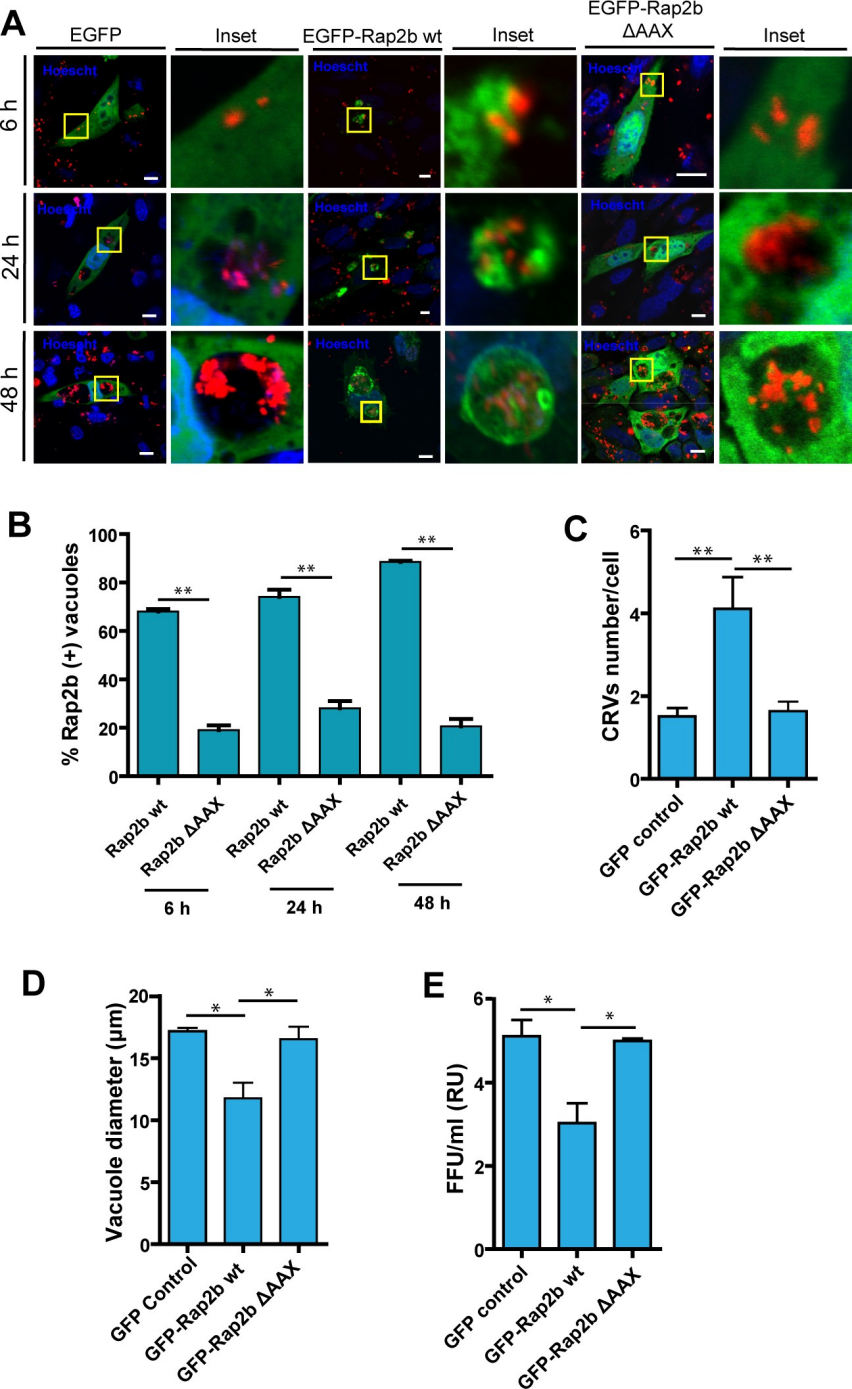
GFP-Rap2b associates with the *C*. *burnetii* vacuole and alters the CRV normal development. A. CHO cells were transfected with pGFP, wt pEGFP-Rap2b or pEGFP-Rap2b ΔAAX and infected with mCherry-*C*. *burnetii* (red) for 6, 24 and 48 h, fixed, and processed by fluorescence microscopy (MOI = 10). B. Quantification of the percentage of wt pEGFP-Rap2b positive vacuoles. C. Quantification of the amount of vacuoles in CHO cells overexpressing GFP (controls), wt pEGFP-Rap2b or the mutant pEGFP-Rap2b ΔAAX at 48 hours after infection with *C*. *burnetii*. D. Quantification of the diameter of the vacuoles in CHO cells overexpressing the constructs indicated above, at 48 hours after infection with *C*. *burnetii*. The data represent the mean ± SEM of at least 3 independent experiments. Tukey test ** p ≤ 0.01; * p ≤ 0.05. E. CHO cells were infected for 48 h with *C*. *burnetii* to allow the development of the *Coxiella* vacuole. The cells were then lysed by sonication and the supernatant was diluted (1:100) and used to infect Vero cells. After 72 h of incubation (chase), cells were fixed and examined by fluorescence microscopy. Data represent the mean ± SEM of at least three independent experiments where a minimum of 1,000 cells were scored in each experiment (*p ≤0.05.). Bars = 10μm.

In order to validate whether the EPAC-Rap2b pathway regulates *C*. *burnetii* infection, we analyzed the role of Rap2b in *C*. *burnetii* infected cells. Thus, CHO cells transiently over-expressing wt EGFP-Rap2b were infected with *C*. *burnetii* and 6, 24, and 48 h p.i., cells were fixed and analyzed by LSCM. As shown in Fig 2A, wt EGFP-Rap2b is recruited at the CRV membrane from early p.i times. About 70–80% of the *Coxiella*-containing phagosomal compartments are labeled with Rap2b (Fig 2B).

To further analyze the recruitment of Rap2b to the CRV, we employed the mutant version of Rap2b, EGFP-Rap2b ΔAAX. This mutant bears a deletion in its C-terminal and cannot be lipidated. As a consequence, it does not localize to the plasma membrane being biologically inactive. CHO cells transiently over-expressing EGFP-Rap2b ΔAAX were infected with *C*. *burnetii* and at 6, 24 and 48 h p.i. cells were fixed and analyzed by LSCM (Fig 2A). As expected, in infected cells, the mutant version of Rap2b was not recruited to the CRV. Fig 2B shows the percentage of vacuoles that recruited Rap2b wt and the ΔAAX mutant, clearly indicating that the CAAX motif is critical for the association of Rap2b with the *Coxiella* containing vacuoles at 6, 24 and 48 h p.i. About 70–80% of the *Coxiella*-containing phagosomal compartments are labeled with wt Rap2b.

### The over-expression of Rap2b wt alters the normal development of the CRV

Upon analyzing the recruitment of Rap2b to CRV in cells transiently over-expressing GFP-Rap2b, we observed that, the diameter of the CRVs was decreased, while the number of *C*. *burnetii* vacuoles was considerably high (Fig 2C and Fig 2D). These results suggest that the normal development of the CRV is affected by the over-expression of wt EGFP-Rap2b. To assess the role of this protein in bacteria replication, we performed FFU assays employing CHO cells transiently over-expressing either Rap2b wt or the inactive mutant Rap2b ΔAAX, and infected with *C*. *burnetii*. As shown in Fig 2E, a marked decrease in the replicative capacity of *C*. *burnetii* was observed in cells transiently over-expressing Rap2b wt, in comparison with cells transfected with GFP and Rap2b ΔAAX mutant. Taken together these results strongly suggest the existence of an inhibitory effect on the functional Rap2b protein during *C*. *burnetii* replication.

### siRNA-mediated knockdown of EPAC and Rap2b favors CRV development

**Fig 3:**
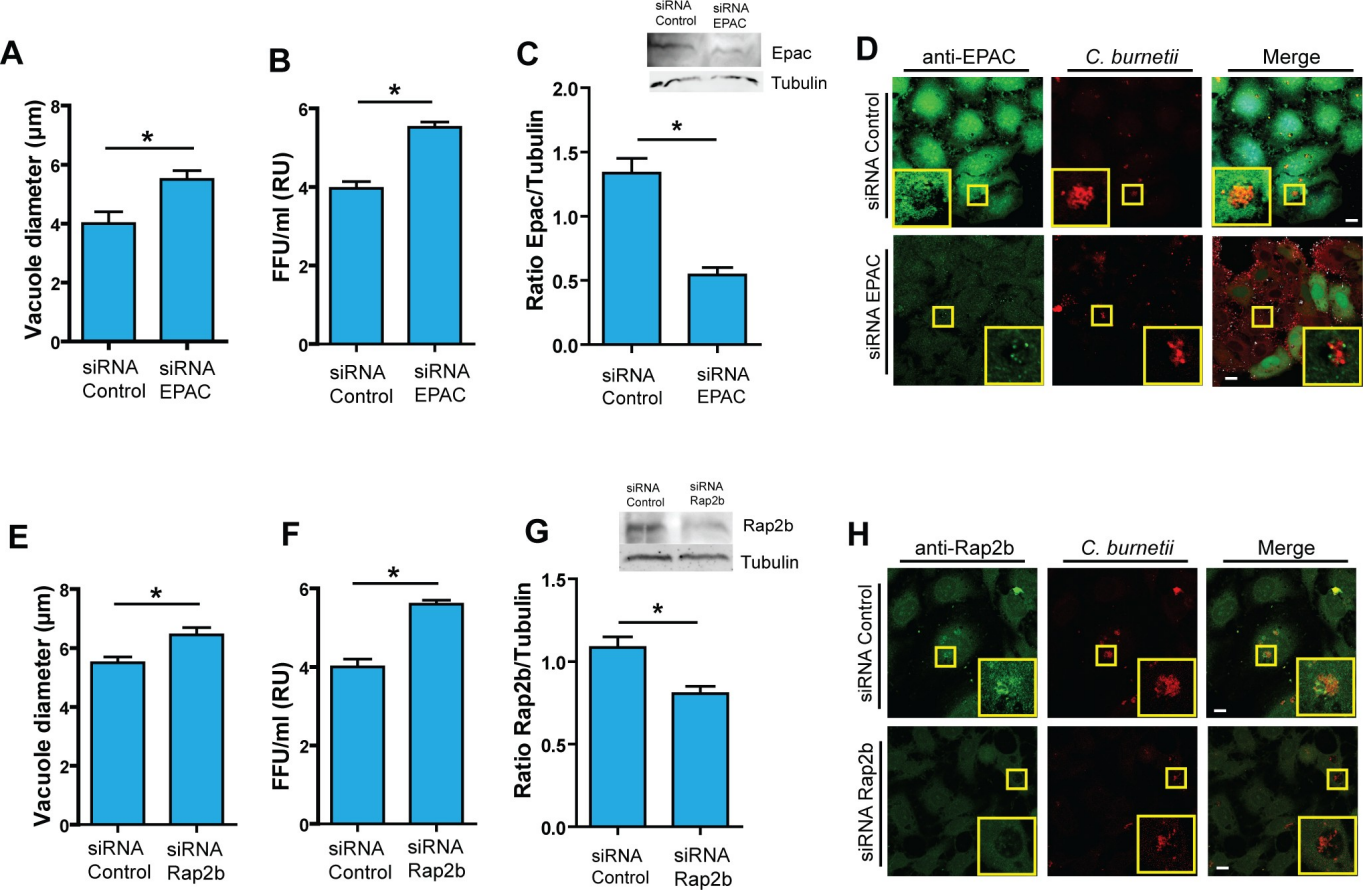
The knockdown of EPAC or Rap2b alters development of the *Coxiella* vacuole. **A.** Hela cells were transfected with siRNA against EPAC or an irrelevant siRNA as negative control. After 48 h, cells were transfected for a second time, infected with *C*. *burnetii* and cultured for an additional 48 h period to allow the development of the large *C*. *burnetii* vacuole (MOI = 10). Subsequently, cells were fixed and subjected to indirect immunofluorescence for the detection of both EPAC and *Coxiella* using specific antibodies. Images were captured by confocal microscopy. Quantification of the vacuole diameter in cells depleted for EPAC compared with the control condition. Data represent the mean ± SEM of at least three independent experiments in which at least 200 vacuoles were scored in each experiment. p≤0.01 Data represent the mean ± SEM of at least two independent experiments. **B.** HeLa cells were transfected with siRNA against EPAC or an irrelevant siRNA as negative control. After 48 h, cells were transfected for a second time, infected with *C*. *burnetii* and cultured for an additional 48 h period to allow the development of the large *C*. *burnetii* vacuole. The cells were then lysed by sonication and the supernatant was diluted (1:100) and used to infect Vero cells. After 72 h of incubation (chase), cells were fixed and examined by fluorescence microscopy. Data represent the mean ± SEM of at least three independent experiments where a minimum of 1,000 cells were scored in each experiment (*p ≤0.05.). **C.** Western blot of the assay described in A and quantification of intensity of the EPAC bands relative to tubulin. **D.** HeLa cells were transfected with a siRNA against EPAC or an irrelevant siRNA as negative control. After 48 h, cells were transfected for a second time, infected with *C*. *burnetii* and cultured for an additional 48 h period. Subsequently, cells were fixed and subjected to indirect immunofluorescence for the detection of both EPAC and *Coxiella* using specific antibodies. Images were captured by confocal microscopy. The panels show that in cells treated with siRNA there is an absence of EPAC labeling in cells indicating that EPAC was effectively depleted. **E**. HeLa cells were transfected with siRNA against Rap2b or an irrelevant siRNA as negative control. After 48 h, cells were transfected for a second time, infected with *C*. *burnetii* and cultured for an additional 48 h period to allow the development of the large *C*. *burnetii* vacuole (MOI = 10). Subsequently, cells were fixed and subjected to indirect immunofluorescence for the detection of both Rap2b and *Coxiella* using specific antibodies. Images were captured by confocal microscopy. Quantification of the vacuole diameter in cells depleted for Rap2b compared with the control condition. Data represent the mean ± SEM of at least three independent experiments in which at least 200 vacuoles were scored in each experiment. p≤0.01 Data represent the mean ± SEM of at least two independent experiments. **F.** HeLa cells were transfected with siRNA against Rap2b or an irrelevant siRNA as negative control. After 48 h, cells were transfected for a second time, infected with *C*. *burnetii* and cultured for an additional 48 h period to allow the development of the large *C*. *burnetii* vacuole. The cells were then lysed by sonication and the supernatant was diluted (1:100) and used to infect Vero cells. After 72 h of incubation (chase), cells were fixed and examined by fluorescence microscopy. Data represent the mean ± SEM of at least three independent experiments where a minimum of 1,000 cells were scored in each experiment (*p ≤0.05.). **G.** Western blot of the assay described in D and quantification of intensity of the Rap2b bands relative to tubulin. **H.** HeLa cells were transfected with siRNA against Rap2b or an irrelevant siRNA as negative control. After 48 h, cells were transfected for a second time, infected with *C*. *burnetii* and cultured for an additional 48 h period. Subsequently, cells were fixed and subjected to indirect immunofluorescence for the detection of both Rap2b and *Coxiella* using specific antibodies. Images were captured by confocal microscopy. The panels show that in cells treated with siRNA there is an absence of Rap2b labeling in cells indicating that Rap2b was effectively depleted.

In order to corroborate the regulatory role of a functional EPAC and Rap2b in the development of the CRV, knockdown assays with specific siRNAs were performed. We designed a double hit protocol in order to ensure the silencing of either EPAC or Rap2b. HeLa cells were grown in 6-well dishes with a small coverslip in each well. Cells were then transfected with a siRNA against EPAC or an unrelated siRNA as control. After 48h post-transfection (p.t.) cells were transfected again and then infected with *C*. *burnetii* for 48 h. The cells in the well were then processed for a FFU assay. As shown in Fig 3A and 3D, the EPAC silencing caused an increase in the CRVs dimensions. Furthermore, a significantly increase in the replicative capacity of *C*. *burnetii* was observed in EPAC depleted cells in comparison with cells transfected with the control siRNA (Fig 3B).

To evaluate the participation of Rap2b, Hela cells were grown, as explained above, in 6-well dishes with a small coverslip in each well. Cells were transfected with a siRNA against Rap2b or an unrelated siRNA as control. After 48 h p.t., cells were transfected again and infected with *C*. *burnetii* for 48 h. Cells were then processed for a FFU assay. By Western blot assay, the silencing of EPAC and Rap2b by the siRNA treatment was demonstrated (Fig 3C and Fig 3G). As shown in Fig 3E, Rap2b silencing caused an increase in the CRVs size. Also, a significantly increase in the replicative capacity of *C*. *burnetii* was observed in cells depleted in Rap2b, in comparison with a siRNA control transfected cells (Fig 3F). This result clearly indicates that Rab2b exerts an inhibitory effect in vacuole development which is consistent with the observed effect when the function of Rap2b was affected by Rap2b overexpression.

### The recruitment of EPAC and Rap2b depends on *C*. *burnetii* secretion system T4SS

**Fig 4:**
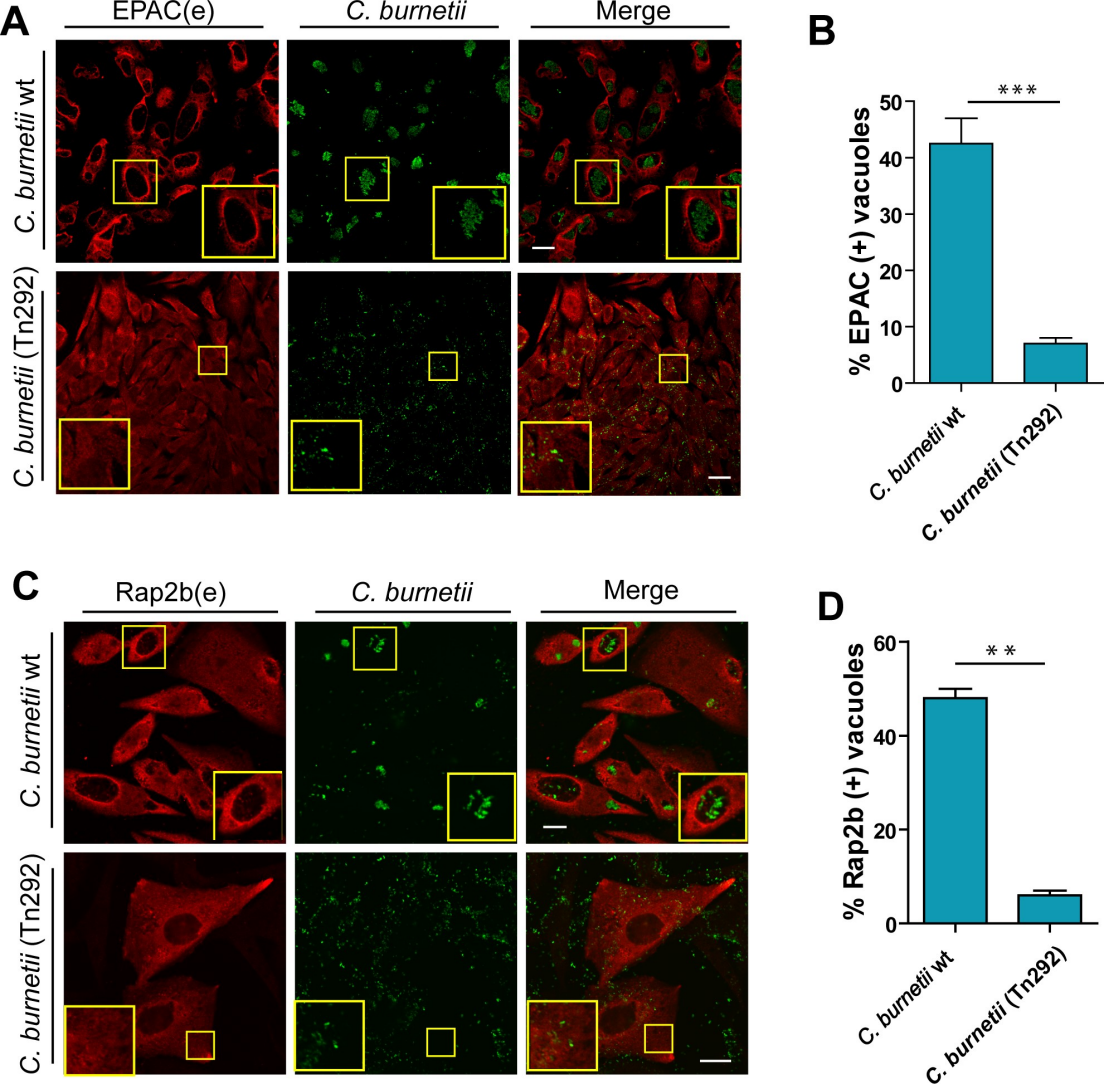
EPAC and Rap2b recruitment is dependent on the bacterial secretion system. **A.** CHO cells were infected with GFP-*C*. *burnetii* and GFP*-C*. *burnetii* DotA mutant (Tn292) (MOI = 10). After 48 h of infection, cells were fixed and analyzed by immunofluorescence using specific antibodies against EPAC (red). Cells were then analyzed by confocal microscopy. **B.** Percentage of CRVs labeled with EPAC from images like the ones depicted in panel A. Data are expressed as mean ± S.E.M. of at least three independent experiments (n>50 cells/group). **C.** CHO cells were infected with GFP-*C*. *burnetii* and GFP*-C*. *burnetii* DotA mutant (Tn292). After 48 h of infection, cells were fixed and analyzed by immunofluorescence using specific antibodies against Rap2b (red). Cells were then analyzed by confocal microscopy. **D.** Percentage of CRVs labeled with Rap2b from images like the ones depicted in panel A. Data are expressed as mean ± S.E.M. of at least three independent experiments (n>50 cells/group). Scale bar: 10 μm. Scale bar: 10 μm.

It has been shown that *C*. *burnetii* has a T4SS termed Dot/Icm. Recent studies have demonstrated that the Dot/Icm functions are required for cytosolic delivery of numerous substrates, identified as proteins effectors (Newton and Roy, 2011; Beare et al., 2012). Since the recruitment of EPAC/Rap2b to the CRV could be induced by these protein effectors, we studied the dependence of T4SS signals on the recruitment of EPAC and Rab2b to the CRV. For this purpose, CHO cells were infected with either wt GFP-*C*. *burnetii* or a DotA mutant GFP-*C*. *burnetii* (Tn292), a mutant that carries an independent transposon insertion in the CBU_1648, which encodes DotA, an essential component of the *Coxiella* Dot/Icm secretion system. At 48 h p.i., cells were fixed and processed for immunofluorescence to detect EPAC and Rap2b. Since EPAC and Rap2b were not recruited to the CRV of the DotA mutant, GFP-*C*. *burnetii* (Tn292), we concluded that this recruitment is dependent on the *C*. *burnetii*’s Dot/Icm secretion system (Fig 4A and Fig 4C). Significant differences were found between both conditions as regards the percentage of recruitment (Fig 4B and Fig 4D).

### The overexpression of Rap2b wt alters both, heterotypic and homotypic fusions with the *C*. *burnetii* vacuoles, at longer times of infection

The CRV has characteristics of a phagolysosome compartment and is extremely fusogenic, recruiting components of the fusion machinery such as the SNAREs Vamp7, Vamp8 and Vamp3 [7]. The enlargement of the *C*. *burnetii* vacuole is achieved by both the homotypic fusion between *Coxiella* phagosomes and the heterotypic fusion between *Coxiella* phagosomes and lysosomes or other cellular compartments.

**Fig 5:**
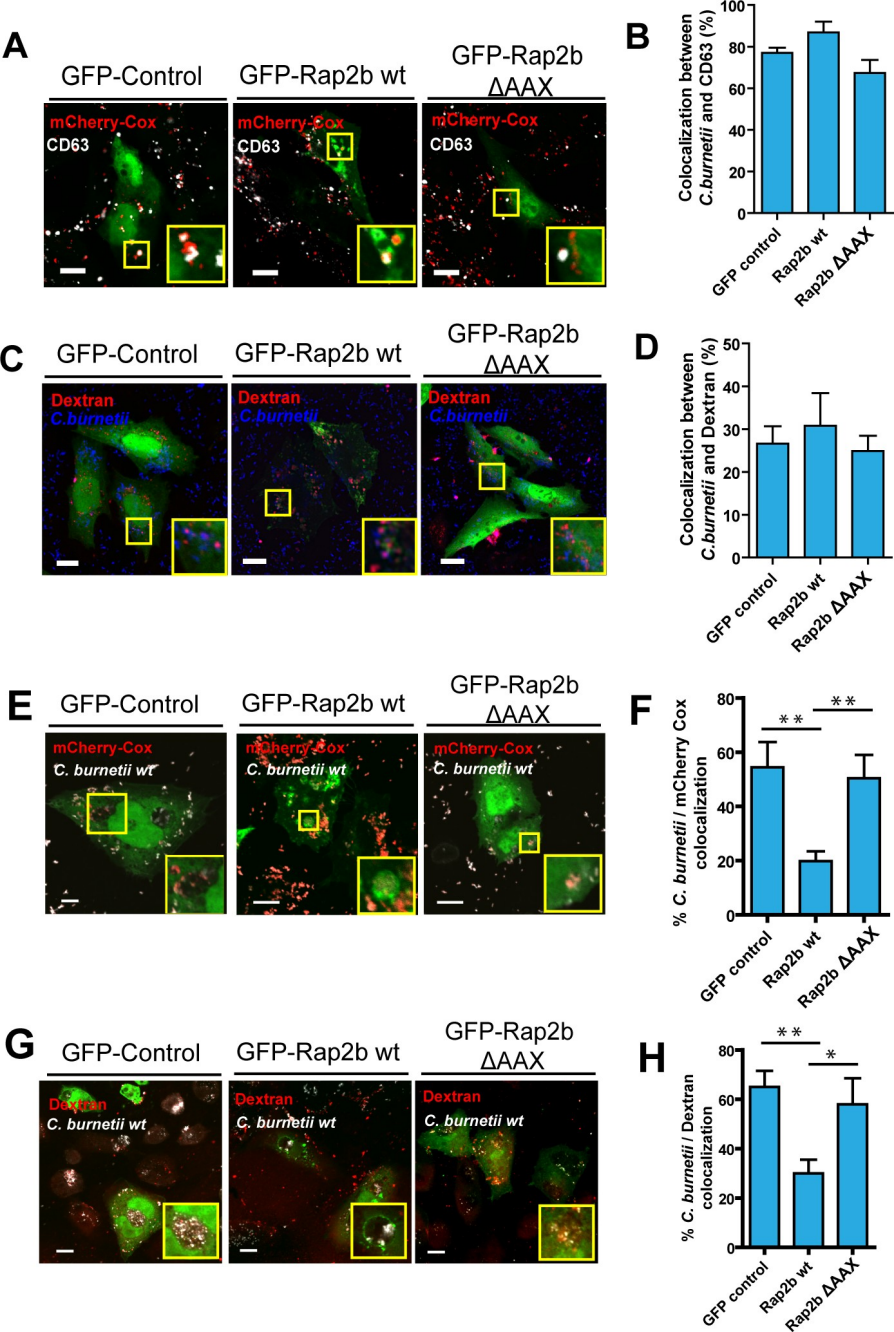
Homotypic fusion between phagosomes containing *C*. *burnetii* and the replicative vacuole is altered by overexpression of Rap2b wt at later times of infection. **A.** CHO cells were transfected with pEGFP, wt pEGFP-Rap2b or pEGFP-Rap2b ΔAAX. After 24 hours the cells were infected with mCherry-*C*.*burnetii* for 2 hours, fixed and subjected to indirect immunofluorescence using antibodies against CD63 (MOI = 10). **B.** Quantification of colocalization between *C burnetii* and CD63. **C.** CHO cells were transfected with pEGFP (control), wt pEGFP-Rap2b or pEGFP-Rap2b ΔAAX and were incubated for 2 hours with 5 μg/ml dextran-rhodamine to allow the labeling of lysosomes. After 24 hours, the cells were infected with *C*. *burnetii* for 2 h, fixed and subjected to indirect immunofluorescence using antibodies against *C*. *burnetii* (blue). **D.** Quantification of colocalization between dextran and *C*. *burnetii*. The data represent the mean ± SEM of at least 3 independent experiments. Tukey test * p ≤ 0.05. Bars = 10 μm. **E.** CHO cells were infected with *C*. *burnetii* for 24 hours and then transfected with pEGFP, pEGFP-Rap2b or pEGFP-Rap2b ΔAAX. After 24 hours, cells were infected with *C*. *burneti* mCherry for 4 h (early phagosomes), fixed and analyzed by confocal microscopy. **F.** Quantification of the arrival of mCherry-*C*. *burnetii* (red) to the vacuole of *C*. *burnetii* already formed. **G.** CHO cells were infected with *C*. *burnetii* for 24 h and then transfected with pEGFP, wt pEGFP-Rap2b or pEGFP-Rap2b ΔAAX. After 24 hours, cells were incubated with Texas red dextran for 4 h, fixed and subjected to indirect immunofluorescence using antibodies against *C*. *burnetii* (white). **H.** Quantification of the arrival of Texas red dextran (red) to the vacuole of *C*. *burnetii* already formed. The data represent the mean ± SEM of at least 3 independent experiments. Tukey test **p≤0.01, * p ≤0.05. Bars = 10 μm.

Since in *C*. *burnetii* infected cells overexpressing Rap2b wt we observed an increase in the number of vacuoles and a reduction in their size, we hypothesized that the over-expression of Rap2b wt could impair the capacity to fuse with other compartments, such as late endocytic vesicles, thus interfering with the normal development of CRVs. Hence, we analyzed the role of Rap2b in the fusion events taking place at early and late p.i times. First, to evaluate the heterotypic fusion process, the presence of the protein CD63 was assessed in late endosomes/lysosomes. CHO cells transiently over-expressing pEGFP, wt pEGFP-Rap2b or pEGFP-Rap2b ΔAAX were infected with mCherry-*C*.*burnetii* for 2 h to analyze early *C*. *burnetii* phagosomes. Cells were then fixed and processed for indirect immunofluorescence to detect CD63 and analyzed by LSCM. Non-significant differences were observed in the CD63 localization levels on the phagosomes containing *C*. *burnetii* between cells transiently over-expressing pEGFP, wt pEGFP-Rap2b or pEGFP-Rap2b ΔAAX (Fig 5A and Fig 5B). It can be concluded that Rap2b did not affect the heterotypic fusion events with the *C*. *burnetti* taking place at early p.i. times. To corroborate these results about the role of Rap2b in heterotypic fusion events we analyzed the fusion of early *C*. *burnetii* phagosomes with dextran pre-loaded lysosomes. It is known that molecules internalized by fluid phase endocytosis (e.g, dextran) can reach the vacuoles containing *C*. *burnetii*. Thus, CHO cells were incubated with 5 μg/ml of dextran-rhodamine for 2 h and then transfected with pEGFP, wt pEGFP-Rap2b or pEGFP-Rap2b ΔAAX. After 24 h p.t., cells were infected with *C*. *burnetii* for 2 h. Cells were fixed and processed for indirect immunofluorescence using an antibody against *C*. *burnetii* (Fig 5C). Since non-significant differences were observed in heterotypic fusion events in cells over-expressing pEGFP, wt pEGFP-Rap2b or pEGFP-Rap2b ΔAAX, we concluded that Rap2b does not affect heterotypic fusion events between phagosomes containing *C*. *burnetii*, and lysosomes at early p.i times (Fig 5D).

Because the expansion of the *C*. *burnetii* vacuole is accomplished by both heterotypic and homotypic fusion events (i.e. fusion between *Coxiella* phagosomes), the effect of the over-expression of Rap2b in late fusion events, i.e., when the vacuole is fully developed was assessed. First, we analyzed whether Rap2b is involved in homotypic fusion between small phagosomes containing *C*. *burnetii* and the CRV. CHO cells were infected with *C*. *burnetii* and at 24 h p.i. cells were transfected with pEGFP, wt pEGFP-Rap2b or pEGFP-Rap2b ΔAAX. After 24 h, cells were infected with mCherry-*C*. *burnetii* for 4 h and processed for immunofluorescence to detect *C*. *burnetii* (stained in gray) and analyzed by LSCM (Fig 5E). Significant differences were observed in the amount of mCherry-*C*. *burnetii* within the vacuoles, between cells transiently over-expressing Rap2b wt and cells over-expressing GFP alone or the Rap2b ΔAAX mutant (Fig 5F). This result indicates that the over-expression of Rap2b impairs homotypic fusion events between the CRV and *C*. *burnetii* phagosomes at late p.i times. In order to analyze the role of Rap2b in heterotypic fusion events at late p.i. times, CHO cells infected with *C*. *burnetii* for 24 h were transfected with pEGFP, wt pEGFP-Rap2b or pEGFP-Rap2b ΔAAX. After 24 h, cells were incubated with 5 μg/ml of dextran-rhodamine for 4 h and then fixed and analyzed by LSCM. As shown in Fig 5G and Fig 5H, significant differences were observed in the amount of vacuoles with presence of dextran inside between cells overexpressing Rap2b wt, and those transfected with either GFP or Rap2b ΔAAX. Hence, the over-expression of this protein would decrease the heterotypic fusion capacity between vesicles or lysosomes containing dextran, which is indicative that heterotypic fusion is affected by overexpression of Rap2b wt at later times of infection. Furthermore, as expected, in CHO cells transiently over-expressing the mutant version of Rap2b, EGFP-Rap2b ΔAAX, homotypic and heterotypic fusion events were not affected at later p.i. times, indicating that it is likely that fusion with other compartments remains intact in cells overexpressing this mutant but not the wt active Rap2b.

### The overexpression of Rap2b wt increases the association of LC3 to the *C*. *burnetii* replicative compartment by increasing the processing of LC3-I to LC3-II

**Fig 6:**
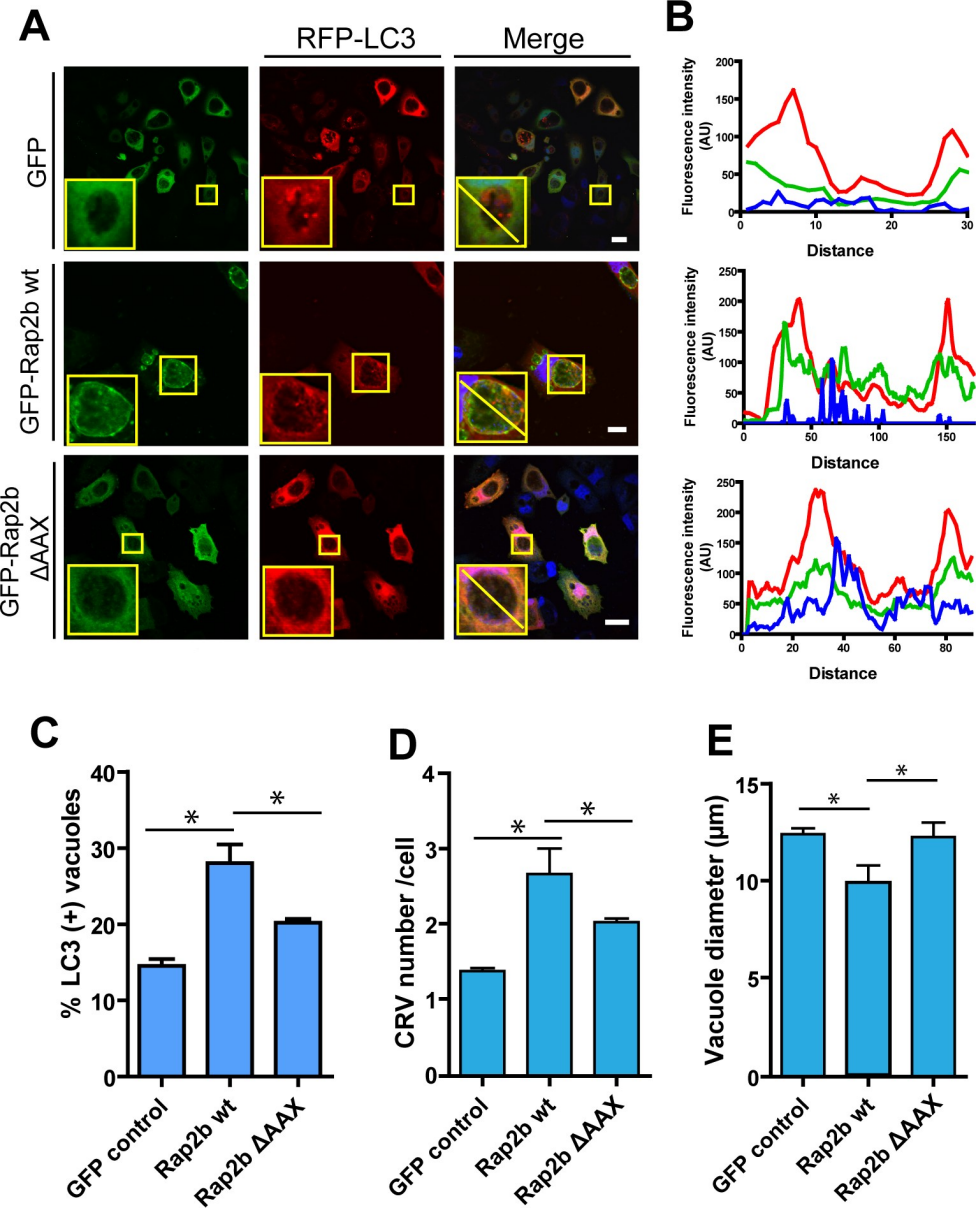
Overexpressed Rap2b increases the recruitment of LC3 to the *C*. *burnetii* vacuole. **A.** CHO cells were co-transfected with RFP-LC3 and pEGFP (control), wt pEGFP-Rap2b or pEGFP-Rap2b ΔAAX and subsequently infected with *C*. *burnetii* for 48 hours (MOI = 10). Cells were fixed and processed for fluorescence microscopy. **B.** Fluorescence intensity along the yellow line depicted in the insets of panel A. **C.** Quantification of the number of LC3 vacuoles in cells transfected with the indicated constructs in A. **D.** Quantification of the vacuole number in cells transfected with the indicated constructs. **E.** Quantification of the vacuole diameter in cells transfected with the indicated constructs. The data represent the mean ± SEM of at least 2 independent experiments. Tukey test * p ≤ 0.05. Bars = 10 μm.

We have previously demonstrated that the activation of autophagy favors the development of the CRV and the bacterial growth [3][4] [28]. Moreover, it is also known that the inhibition of autophagy induced by high intracellular levels of cAMP is mediated by EPAC and Rap2b [22]. On the other hand, we have also found that Rap2b inhibits the autophagic response induced by the *S*. *aureus* α-hemolysin (Hla) [23]. In order to determine the connection between Rap2b and the autophagy pathway in *C*. *burnetti* infected cells, CHO cells were co-transfected with pEGFP-Rap2b and the autophagic marker RFP-LC3 and at 24 h p.t., they were infected with *C*. *burnetii* for 48 h. Finally, cells were fixed and analyzed by LSCM. As depicted in Fig 6A and Fig 6B, a recruitment of RFP-LC3 protein to a population of the CRVs was observed in GFP transfected control cells in cells over-expressing Rap2b wt and in cells transfected with the Rap2b mutant. However, an increased percentage of vacuoles recruiting LC3 were observed in cells transfected with Rap2b wt (Fig 6C). The number of CRV was also determined in cells transfected with Rap2b wt and Rap2b ΔAAX and infected with *C*. *burnetti* after 24 h p.t. Cells were fixed, processed for fluorescence microscopy and evaluated by LSCM. Consistent with our previous observations, an increased number of vacuoles with a slightly smaller size were observed in cells overexpressing Rap2b wt, as compared to the EGFP control (Fig 6D and Fig 6E). A bigger number of vesicles was also observed in cells over-expressing the mutant Rap2b ΔAAX, but in this case, no significant changes in the size of vacuoles, were observed. The moderate decrease in the size of the CRVs induced by the expression of Rab2b could be attributed to the over-expression of LC3 alone, which proved to increase the size and development of the CRV. Therefore, the co-expression of this protein may compensate the effect of wt Rab2b on the decrease in vacuole size and the consequent increase in number.

**Fig 7:**
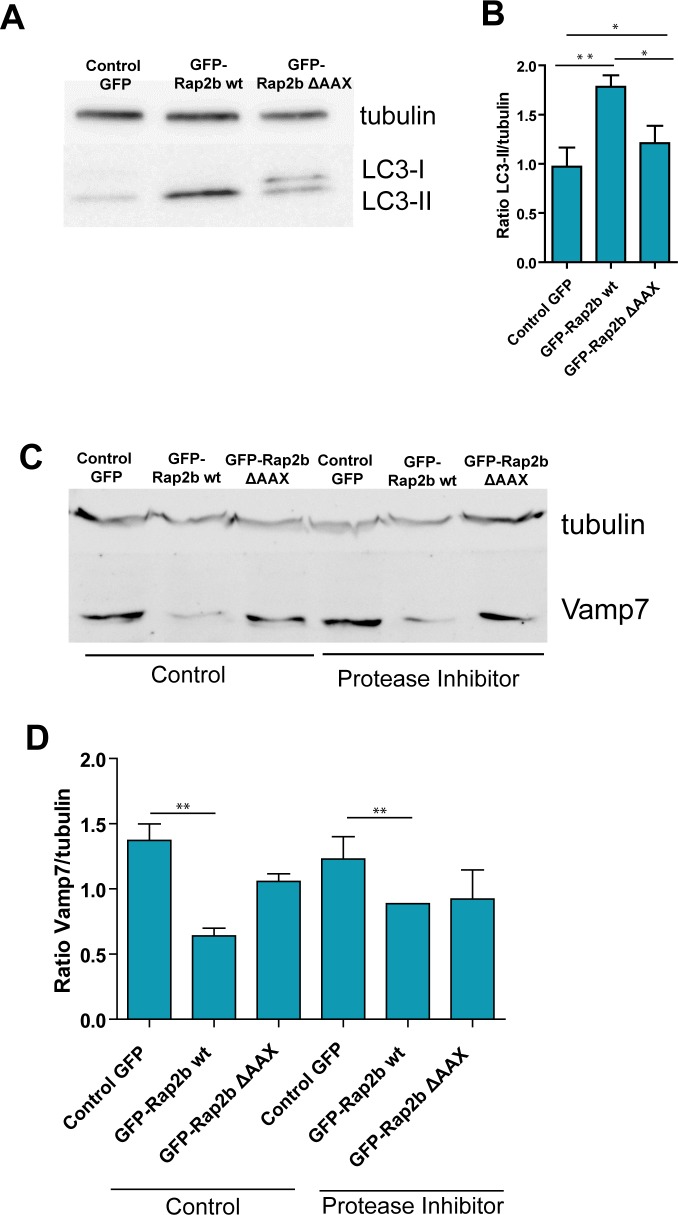
LC3 and Vamp7 are altered by overexpression of Rap2b wt in control and infected CHO cells. **A.** CHO cells were transfected with pEGFP, wt pEGFP-Rap2b or pEGFP-Rap2b ΔAAX. After 24 hours, cells were incubated with *C*. *burnetii* mCherry for 48 h (MOI = 10). Afterwards, cells were lysed with sample buffer and the samples were subjected to Western blot analysis using a rabbit anti-LC3 and the corresponding HRP-labeled secondary antibody, and subsequently developed with an enhanced chemiluminescence detection kit. These data are representative of three independent experiments. **B.** Quantification of the intensity of the LC3-II bands relative to tubulin from the Western blot of the assay described in A. The data represent the mean ± SEM of at least 3 independent experiments. Tukey test **p≤0.01, *p ≤0.05. **C.** CHO cells were transfected with pEGFP, wt pEGFP-Rap2b or pEGFP-Rap2b ΔAAX. After 24 hours, cells were incubated with *C*. *burnetii* mCherry for 48 h in the presence of complete medium without (Control) or with protease inhibitors. Afterwards, cells were lysed with sample buffer and the samples were subjected to Western blot analysis using a mouse anti-Vamp7 and the corresponding HRP-labeled secondary antibody, and subsequently developed with an enhanced chemiluminescence detection kit. These data are representative of three independent experiments. **D.** Quantification of the intensity of the Vamp7 bands relative to tubulin in the Western blot of the assay described in E. The data represent the mean ± SEM of at least 3 independent experiments. Tukey test **p≤0.01, * p ≤0.05.

Taken together our results suggest that the overexpression of Rap2b wt may alter the autophagic response of the host cell to *C*. *burnetii* infection. To corroborate these results, the processing of LC3 was analyzed by Western blot in CHO cells transiently transfected with pEGFP, wt pEGFP-Rap2b or pEGFP-Rap2b ΔAAX, and infected with *C*. *burnetii* for 48 h (Fig 7A). As shown in Fig 7B, significantly increased levels of lipidated LC3 protein (LC3-II) was observed in cells transfected with Rap2b wt. On the other hand, the levels of LC3-II in cells transiently over-expressing Rap2b ΔAAX remained unchanged.

### Over-expression of Rap2b wt affects the intracellular levels of the SNARE protein Vamp7

Taking into account that overexpression of Rap2b wt alters both, heterotypic and homotypic fusions with the *C*. *burnetii* vacuoles, at later times of infection, we were interested in assessing the possible mechanism involved at the molecular level. Vamp7 is a SNARE protein that participates in heterotypic and homotypic fusion with the CRVs [7]. Indeed, we have previously demonstrated that *C*. *burnetti* is able to dynamically recruit Vamp7 in order to promote fusion with vesicles of the endocytic-lysosomal pathway to stimulate the CRV growth. Also, we have demonstrated that Vamp7 is important for homotypic fusions which also contributes to the vacuole development [7]. We therefore investigated the effect of the overexpression of Rap2b in the levels of Vamp7. Thus, CHO cells were transiently transfected with pEGFP, wt pEGFP-Rap2b or pEGFP-Rap2b ΔAAX and after 24 h p.t. cells were infected with *C*. *burnetii* for 48 h. As shown in Fig 7C and Fig 7D, a significant reduction of the intracellular levels of Vamp7 protein was observed in cells transfected with wt pEGFP-Rap2b whereas no major effects in the Vamp7 levels were observed when the truncated mutant Rap2b ΔAAX was overexpressed.

In order to rule out the possibility that the reduction in intracellular levels of Vamp7 was due to lysosomal degradation, CHO cells were transfected with either pEGFP, wt pEGFP-Rap2b or -Rap2b ΔAAX and after 24 h p.t., cells were incubated in complete culture medium supplemented with protease inhibitors (leupeptin, E64d and pepstatin A) or with DMSO as control. As shown in Fig 7C and Fig 7D, a significant decrease in the intracellular levels of Vamp7 protein was detected in cells transfected with either wt pEGFP-Rap2b treated with or without protease inhibitors. This observation indicates that the reduction in the levels of Vamp7 observed in Rap2b transfected cells cannot be attributed to lysosomal degradation.

In summary, it can be concluded that the decreased levels of Vamp7 detected in cells overexpressing Rap2b wt may hamper the normal development of the CRVs.

## Discussion

We have previously demonstrated that components of the cAMP/EPAC pathway, such as the protein Rap2b, are recruited to a fraction of phagosomes containing *S*. *aureus* and that this protein inhibits the host´s autophagic response induced by the *S*. *aureus* α-toxin [23]. EPAC, a cAMP-dependent protein functions as a GEF (nucleotide exchange factor) of the Rap GTPases. We have observed that EPAC associates with the vacuole of *C*. *burnetii* at later times post infection (48 h). Interestingly, when infected cells were treated with 8-pCPT-cAMP, an EPAC specific activator, a considerable decrease in the vacuole size was observed. More importantly, treatment with 8-pCPT-cAMP altered the replicative capacity of *C*. *burnetii*. Consistent with this observation, silencing of this protein markedly increased the CRV size as well as the replication of *C*. *burnetii*, suggesting that activation of EPAC may exert an inhibitory effect in both vacuole development and bacterial growth. In our current study, we have also found that the over-expression of EGFP-Rap2b, but not Rap1b (data not shown) in infected cells, affects the normal development of the CRV indicated by the decrease in vacuolar size and number of bacteria as determined by FFU assays. In agreement with this inhibitory effect, we have shown that knocking down Rap2b resulted in an increase in bacterial growth. Several studies have shown the association of Rap2 with Rab2, a small GTPase involved in Golgi-endoplasmic reticulum vesicle transport [29] and that Rap2 proteins are essential to the recycling endosomal membrane, in a palmitoylation-dependent manner [30]. Rap2a has been localized to the Golgi complex [29] and it has also been specifically involved in the establishment of polarity in intestinal epithelial cells [31]. However, the distinct function of the three Rap2 isoforms (Rap2a, Rap2b, and Rap2c) has not been determined [31] [32]. Thus, further studies are necessary to address whether the inhibitory effect of Rap2b may be related to other transport events.

It is known that the CRV is highly fusogenic with either other phagosomes containing *C*. *burnetii* (homotypic fusion) or vesicles from the endocytic, autophagic and secretory pathways (heterotypic fusion). As demonstrated before, an inhibition of fusion causes a decrease in the size of the CRVs and therefore increases the number of smaller vesicles containing *C*. *burnetii* [7]. Since over-expression of Rap2b causes a marked increase in the number of vacuoles but with a concomitant decrease in size, we postulated that fusion events were altered. Indeed, we have shown that expression of Rap2b wt reduced both, the homotypic and the heterotypic fusion capacity of the CRV. However, this inhibitory effect was somehow altered by co-expression with GFP-LC3. Previously studies indicate that the presence of LC3 in the membrane of phagosomes promotes fusion with lysosomes in a process known as phagocytosis associated to LC3 (LAP) [33]. The presence of LC3 in the membrane of the CRV may facilitate the vacuole to fuse with other vesicles and lysosomes. This could explain the increase in the size of the vacuoles in infected cells overexpressing together Rap2b and LC3, since the presence of overexpressed LC3 would counteract the Rap2b inhibitory effect.

This study, in line with other works, demonstrates that the relationship between the CRV and the autophagic pathway is important for the CRV development and the fusion with autophagosomes [7][34][35]. More importantly, the effects we observed occurred at later times of infection, when the large replicative vacuole is already formed. This finding would explain the differences observed at either early or late times of post-infection, since it is at later times when the vacuole requires more membrane from other compartments. According to recent studies of Voth et al, these interactions with autophagosomes taking place after 24 h of infection are indispensable [36]. In addition, recent studies have revealed the existence of interactions between a *Coxiella* effector called Cig2 and the autophagic pathway [37]. The multivacuolar phenotype of cells infected with the Cig2::Tn mutants are similar to that of cells with Stx17 silenced and infected with the strain NMII. The Cig2 mutant of *C*. *burnetii* has a multivacuolar phenotype because this effector is important for the control of autophagy by *C*. *burnetii*. Therefore Cig2 would promote the fusion of the CRV with autophagosomes during infection.

In cells overexpressing wt EGFP-Rap2b a decrease was observed in both homotypic and heterotypic fusion events of the CRV at later times of infection (i.e. 48 h), suggesting that Rap2b wt acts mainly at later times during the infection. Moreover, the hampering of fusion events between the CRV and other vesicles have also been observed in v-SNAREs deficient cells [7][38]. We have previously demonstrated that the silencing of the protein Vamp7 causes a decrease in (both homotypic and heterotypic fusion, as the vacuole of *C*. *burnetii* was not able to fuse with lysosomes or with other CRV in the absence of Vamp7 [7][39]. Since *C*. *burnetii* promotes the generation of highly fusogenic phagosomes, we studied the contribution of Vamp7 in the generation and development of *C*. *burnetii-*containing vacuoles in cells over-expressing Rap2b. We determined the intracellular levels of Vamp7, which is involved in the heterotypic fusion of late endosomes and lysosomes and, strikingly, low levels of Vamp7 in cells overexpressing Rap2b wt were observed. Since Vamp7 is not affected by the presence of proteases inhibitors, it can be inferred that the decreased levels of Vamp7 protein in cells overexpressing Rap2b wt is not due to lysosomal proteolysis.

In conclusion, in this report we have demonstrated that the cAMP effectors EPAC and Rap2b are key regulators of CRV development. We have proved that the small GTPase Rap2b has a critical role in the development of CRV by altering the fusion events of the *Coxiella* vacuole and that this effect might be at least in part, by modulating the levels of Vamp7. This is a very intriguing observation. However, further studies would be necessary to determine the critical molecular mechanisms involved in this modulation.

## Supporting information

S1 Table Experimental CRV measures. (PDF)

## References

[1] Voth DE, Heinzen RA. Lounging in a lysosome: The intracellular lifestyle of Coxiella burnetii. Cellular Microbiology. 2007 pp. 829–840. 10.1111/j.1462-5822.2007.00901.x 17381428

[2] Gutierrez MG, Vázquez CL, Munafó DB, Zoppino FCM, Berón W, Rabinovitch M, et al Autophagy induction favours the generation and maturation of the Coxiella-replicative vacuoles. Cell Microbiol. 2005;7: 981–93. 10.1111/j.1462-5822.2005.00527.x 15953030

[3] Berón W, Gutierrez MG, Rabinovitch M, Colombo MI. Coxiella burnetii localizes in a Rab7-labeled compartment with autophagic characteristics. Infect Immun. 2002;70: 5816–21. Available: http://www.pubmedcentral.nih.gov/articlerender.fcgi?artid=128334&tool=pmcentrez&rendertype=abstract 10.1128/IAI.70.10.5816-5821.2002 12228312 PMC128334

[4] Romano PS, Gutierrez MG, Berón W, Rabinovitch M, Colombo MI. The autophagic pathway is actively modulated by phase II Coxiella burnetii to efficiently replicate in the host cell. Cell Microbiol. 2007;9: 891–909. 10.1111/j.1462-5822.2006.00838.x 17087732

[5] Coleman SA, Fischer ER, Howe D, Mead DJ, Heinzen RA. Temporal Analysis of Coxiella burnetii Morphological Differentiation. J Bacteriol. 2004; 7344–7352. 10.1128/JB.186.21.7344-7352.2004 15489446 PMC523218

[6] Weber MM, Chen C, Rowin K, Mertens K, Galvan G, Zhi H, et al Identification of Coxiella burnetii Type IV Secretion Substrates Required for Intracellular Replication and Coxiella-Containing Vacuole Formation. J Bacteriol. 2013;195: 3914–3924. 10.1128/JB.00071-13 23813730 PMC3754607

[7] Campoy EM, Mansilla ME, Colombo MI. Endocytic SNAREs are involved in optimal Coxiella burnetii vacuole development. Cell Microbiol. 2013;15: 922–41. 10.1111/cmi.12087 23217169

[8] Campoy EM, Zoppino FCM, Colombo MI. The early secretory pathway contributes to the growth of the Coxiella-replicative niche. Infect Immun. 2011;79: 402–13. 10.1128/IAI.00688-10 20937765 PMC3019900

[9] Mcdonough JA, Newton HJ, Klum S, Swiss R, Agaisse H, Roy CR. Host Pathways Important for Coxiella burnetii Infection Revealed by Genome-Wide RNA Interference Screening. 2013; 1–13. 10.1128/mBio.00606-12.Editor PMC3560531 23362322

[10] Levine B, Klionsky DJ. Development by self-digestion: molecular mechanisms and biological functions of autophagy. Dev Cell. 2004;6: 463–77. Available: http://www.ncbi.nlm.nih.gov/pubmed/15068787 15068787 10.1016/s1534-5807(04)00099-1

[11] Zoppino FCM, Militello RD, Slavin I, Alvarez C, Colombo MI. Autophagosome formation depends on the small GTPase Rab1 and functional ER exit sites. Traffic. 2010;11: 1246–61. 10.1111/j.1600-0854.2010.01086.x 20545908

[12] Militello RD, Colombo MI. A membrane is born: origin of the autophagosomal compartment. Curr Mol Med. 2011;11: 197–203. Available: http://www.ncbi.nlm.nih.gov/pubmed/21375493 21375493 10.2174/156652411795243441

[13] Pareja MEM, Colombo MI. Autophagic clearance of bacterial pathogens: molecular recognition of intracellular microorganisms. Front Cell Infect Microbiol. FRONTIERS RESEARCH FOUNDATION; 2013;3: 54 10.3389/fcimb.2013.00054 24137567 PMC3786225

[14] Grandoch M, Roscioni SS, Schmidt M. The role of Epac proteins, novel cAMP mediators, in the regulation of immune, lung and neuronal function Abbreviations: 2010; 265–284. 10.1111/j.1476-5381.2009.00458.x 19912228 PMC2825350

[15] Kalamidas SA, Kuehnel MP, Peyron P, Rybin V, Rauch S, Kotoulas OB, et al cAMP synthesis and degradation by phagosomes regulate actin assembly and fusion events: consequences for mycobacteria. J Cell Sci. 2006;119: 3686–94. 10.1242/jcs.03091 16931599

[16] Cherra SJ, Kulich SM, Uechi G, Balasubramani M, Mountzouris J, Day BW, et al Regulation of the autophagy protein LC3 by phosphorylation. J Cell Biol. 2010;190: 533–539. 10.1083/jcb.201002108 20713600 PMC2928022

[17] Roscioni SS, Elzinga CRS. Epac: effectors and biological functions. 2008; 345–357. 10.1007/s00210-007-0246-7 18176800

[18] Branham MT, Bustos M a, De Blas G a, Rehmann H, Zarelli VEP, Treviño CL, et al Epac activates the small G proteins Rap1 and Rab3A to achieve exocytosis. J Biol Chem. 2009;284: 24825–39. 10.1074/jbc.M109.015362 19546222 PMC2757186

[19] Bos JL. Epac proteins: multi-purpose cAMP targets. Trends Biochem Sci. 2006;31: 680–6. 10.1016/j.tibs.2006.10.002 17084085

[20] Pannekoek W-J, Kooistra MRH, Zwartkruis FJT, Bos JL. Cell-cell junction formation: the role of Rap1 and Rap1 guanine nucleotide exchange factors. Biochim Biophys Acta. 2009;1788: 790–6. 10.1016/j.bbamem.2008.12.010 19159611

[21] Kim J-G, Moon M-Y, Kim H-J, Li Y, Song D-K, Kim J-S, et al Ras-related GTPases Rap1 and RhoA Collectively Induce the Phagocytosis of Serum-opsonized Zymosan Particles in Macrophages. J Biol Chem. 2012;287: 5145–5155. 10.1074/jbc.M111.257634 22194606 PMC3281652

[22] Williams A, Sarkar S, Cuddon P, Ttofi EK, Saiki S, Siddiqi FH, et al Novel targets for Huntington’s disease in an mTOR-independent autophagy pathway. Nat Chem Biol. 2008;4: 295–305. 10.1038/nchembio.79 18391949 PMC2635566

[23] Mestre MB, Colombo MI. cAMP and EPAC are key players in the regulation of the signal transduction pathway involved in the α-hemolysin autophagic response. PLoS Pathog. 2012;8: e1002664 10.1371/journal.ppat.1002664 22654658 PMC3359991

[24] Romano PS, Gutierrez MG, Berón W, Rabinovitch M, Colombo MI. The autophagic pathway is actively modulated by phase II Coxiella burnetii to efficiently replicate in the host cell. Cell Microbiol. 2007;9: 891–909. 10.1111/j.1462-5822.2006.00838.x 17087732

[25] Mansilla Pareja ME, Bongiovanni A, Lafont F, Colombo MI. Alterations of the Coxiella burnetii Replicative Vacuole Membrane Integrity and Interplay with the Autophagy Pathway. Front Cell Infect Microbiol. 2017;7: 112 10.3389/fcimb.2017.00112 28484683 PMC5401879

[26] Williams A, Sarkar S, Cuddon P, Ttofi EK, Siddiqi FH, Jahreiss L, et al UKPMC Funders Group Novel targets for Huntington ‘ s disease in an mTOR-independent autophagy pathway. 2009;4: 295–305. 10.1038/nchembio.79.Novel PMC2635566 18391949

[27] Qiao J, Mei FC, Popov VL, Vergara L a, Cheng X. Cell cycle-dependent subcellular localization of exchange factor directly activated by cAMP. J Biol Chem. 2002;277: 26581–6. 10.1074/jbc.M203571200 12000763

[28] Gutierrez MG, Master SS, Singh SB, Taylor G a, Colombo MI, Deretic V. Autophagy is a defense mechanism inhibiting BCG and Mycobacterium tuberculosis survival in infected macrophages. Cell. 2004;119: 753–66. 10.1016/j.cell.2004.11.038 15607973

[29] Pizon V, Desjardins M, Bucci C, Parton RG, Zerial M, U- I, et al Association of Rap1a and Rap1b proteins with late endocytic / phagocytic compartments and Rap2a with the Golgi complex. 1994;1670: 1661–1670. 10.1242/jcs.107.6.1661 7962206

[30] Uechi Y, Bayarjargal M, Umikawa M, Oshiro M, Takei K, Yamashiro Y, et al Rap2 function requires palmitoylation and recycling endosome localization. Biochem Biophys Res Commun. Elsevier Inc.; 2009;378: 732–737. 10.1016/j.bbrc.2008.11.107 19061864

[31] Bruurs LJM, Bos JL. Mechanisms of isoform specific Rap2 signaling during enterocytic brush border formation. PLoS One. Public Library of Science; 2014;9: e106687 10.1371/journal.pone.0106687 25203140 PMC4159233

[32] Mitin N, Rossman KL, Der CJ. Signaling interplay in Ras superfamily function. Curr Biol. 2005;15: R563–74. 10.1016/j.cub.2005.07.010 16051167

[33] Lai S-C, Devenish RJ. LC3-Associated Phagocytosis (LAP): Connections with Host Autophagy. Cells. 2012;1: 396–408. 10.3390/cells1030396 24710482 PMC3901117

[34] Newton HJ, Mcdonough JA, Roy CR. Effector Protein Translocation by the Coxiella burnetii Dot / Icm Type IV Secretion System Requires Endocytic Maturation of the Pathogen-Occupied Vacuole. 2013; 10.1371/journal.pone.0054566 PMC3547880 23349930

[35] Kohler L et al Effector Protein Cig2 Decreases Host Tolerance of Infection by Directing Constitutive Fusion of Autophagosomes with the Coxiella—Containing Vacuole. 2016;7: 1–14. 10.1128/mBio.01127-16.Editor PMC4958265 27435465

[36] Winchell CG, Graham JG, Kurten RC, Voth DE. Coxiella burnetii type IV secretion-dependent recruitment of macrophage autophagosomes. Infect Immun. 2014;82: 2229–38. 10.1128/IAI.01236-13 24643534 PMC4019161

[37] Newton HJ, Kohler LJ, McDonough J a, Temoche-Diaz M, Crabill E, Hartland EL, et al A screen of Coxiella burnetii mutants reveals important roles for Dot/Icm effectors and host autophagy in vacuole biogenesis. PLoS Pathog. 2014;10: e1004286 10.1371/journal.ppat.1004286 25080348 PMC4117601

[38] Mcdonough JA, Newton HJ, Klum S, Swiss R, Agaisse H, Roy CR. Host Pathways Important for Coxiella burnetii Infection Revealed by. 2013; 1–13. 10.1128/mBio.00606-12.Editor PMC3560531 23362322

[39] Fader CM, Sánchez DG, Mestre MB, Colombo MI. TI-VAMP/VAMP7 and VAMP3/cellubrevin: two v-SNARE proteins involved in specific steps of the autophagy/multivesicular body pathways. Biochim Biophys Acta. 2009;1793: 1901–16. 10.1016/j.bbamcr.2009.09.011 19781582

