# Supplementary material for: The cAMP effectors, Rap2b and EPAC, are involved in the regulation of the development of the Coxiella burnetii containing vacuole by altering the fusogenic capacity of the vacuole
Source: PLoS One. 2019 Feb 14;14(2):e0212202. doi: 10.1371/journal.pone.0212202 (PMC6375611; doi:10.1371/journal.pone.0212202)
Supplement: S1 Table — (PDF) [file pone.0212202.s001.pdf]

S1 Table: Experimental CRV measures

Figure 1

Vacuole diameter (um)	
Control	8 pCPT
6,816	9,622
7,29	7,868
5,052	4,04
5,234	4,887
5,856	8,484
8,484	6,661
6,358	7,259
6,184	8,663
14,051	6,503
6,367	13,052
4,704	6,157
5,429	4,598
5,695	5,958
6,157	5,958
13,388	4,239
5,958	3,874
7,327	7,562
5,139	6,04
9,958	6,298
7,951	7,62
10,968	4,976
7,076	4,681
8,631	13,123
4,681	6,04
8,631	7,014
8,374	10,828
6,184	6,661
6,816	4,354
6,184	7,999
5,695	6,751
5,042	3,656
7,972	4,774
8,142	4,887
8,795	5,967
7,349	5,042
4,502	5,306

11,063	7,312
4,704	3,376
5,337	13,656
4,819	14,502
8,688	3,519
5,958	7,468
6,324	3,376
4,646	5,627
3,774	4,976
14,502	4,965
10,132	5,865
6,982	7,62
6,289	3,519
7,349	7,312
9,952	5,695
15,637	8,374
7,229	8,354
5,656	7,548
5,627	7,868
6,982	5,181
5,388	3,052
9,155	5,637
7,29	10,638
8,51	5,781
12,207	7,282
6,824	10,127
4,303	7,972
8,739	5,627
8,301	7,548
6,184	5,139
8,631	5,656
13,792	13,986
6,653	13,701
8,593	8,052
7,161	4,187
8,275	5,921
6,595	4,027
9,802	6,911
6,702	5,042
13,642	3,641
5,429	4,04

7,791	7,951
7,014	4,819
15,009	8,282
14,502	8,992
6,595	6,427
12,513	4,965
10,435	5,865
32,19	5,347
24,75	7,312
13,42	6,009
13,63	7,312
14,92	9,467
8,38	6,367
31,29	8,188
20,9	7,312
29,17	7,312
43,6	4,303
14,17	8,324
8,15	6,04
7,3	4,739
9,26	4,646
11,62	5,388
12,11	13,774
9,16	7,951
10,15	8,53
32,08	7,46
13,78	9,39
16,33	5,388
11,92	8,57
14,13	9,39
8,25	12,7
10,84	8,84
17,82	8,48
23,01	9,01
13,8	5,17
11,428	12,67
10,127	15,12
13,56	13,55
13,35	9,45
13,732	
12,582	

EPAC (+) Vacuoles		
wt-EPAC-GFP	(1-148)-EPAC-GFP	(72-148)-EPAC-GFP
58,71%	61,43%	49,77%
61,74%	58,38%	56,77%
58,71%	59,72%	56,65%
66,36%	41,47%	50,08%
61,19%	50,94%	50,34%
53,53%	58,03%	52,00%
63,55%	67,62%	63,51%
65,94%	58,47%	67,54%
55,30%	67,32%	56,81%
63,43%	53,08%	58,26%
70%	45,19%	52,55%

FFU/ml (RU)		FFU/ml (RU)			
Control	8 pCPT	GFP	wt-EPAC-GFP	(1-148)-EPAC-GFP	(72-148)-EPAC-GFP
3,91869919	1,92857143	4,170940171	1,574162679	4,630952381	3,780346821
3,90654206	1,68627451	3,942857143	1,655813953	4,630573248	4,584337349
3,83333333	1,2125	3,922330097	2,5	4,655844156	3,494791667
3,8041958	1,16666667	4,263157895	2,495049505	3,489932886	3,520958084
3,64285714	0,96428571	3,918181818	1,536458333	4,614285714	3,456410256
3,57462687	1,07865169	4,395833333	2,76419214	4,561151079	4,35
3,86764706	1,72727273	3,861111111	1,50990099	4,671641791	4,420212766
3,77419355	2,05084746	4,137931034	2,440860215	4,598591549	4,357575758
3,00990099	2,88095238	4,054347826	2,460869565	3,666666667	4,399038462
3,24074074	1,73170732	4,066037736	2,610619469	4,60625	
3,90243902	2,40816327	4,361702128	1,549107143	3,680722892	4,592592593
3,36046512		4,235294118	2,519230769	4,61827957	3,459627329
2,8631579	3,27777778	4,185185185		3,597222222	4,685897436
3,01941748		4,205479452	2,530726257	4,654929577	4,369318182
3,9516129		4,166666667		3,739837398	4,62195122
3,27906977	3,02222222			4,787037037	3,405882353
2,85576923	3,25531915	4,298507463	2,833333333	3,907801418	4,413580247
3,33928571		4,734693878	2,608465608	4,705882353	3,690607735
3,90909091	2,19642857	4,113636364	2,610465116	3,869918699	4,543859649
3,93877551	2,68	4,762711864		3,818791946	3,576923077
5				4,084745763	4,724832215
3,95652174					4,615384615

3,48275862	3,48780488		2,657303371	4,886792453	4,732142857
2,97368421	2,62962963		2,758426966		
2,80769231			2,065217391	4,760683761	

Figure 2

Vacuole diameter (um)			CRVs number/Cell		
GFP-Control	GFP-Rap2b wt	GFP-Rap2b CAAX	GFP-Control	GFP-Rap2b wt	GFP-Rap2b CAAX
27,93	5,09	25,51	1	3	2
11,25	8,84	30,465	1	4	2
23,99	23,03	34,028	1	3	1
25,27	9,79	20,535	1	2	2
20,34	13,8	27,295	2	1	2
18,44	8,32	21,987	2	2	3
35,62	5,9	23,59	1	5	2
15,58	17,46	25,707	2	4	1
15,55	10,82	12,459	1	3	2
7,94	3,26	13,166	2	8	3
21,27	4,72	10,674	2	1	2
19,64	8,69	10,061	3	4	1
33,25	8,37	11,135	3	4	1
8,62	7,48	14,651	2	1	1
15,69	8,53	15,038	1	4	2
5,71	7,46	23,527	2	4	2
10,08	13,56	30,245	3	4	2
7,46	13,35	25,389	2	7	2
17,82	8,57	16,891	1	1	2
25,29	9,39	11,958	2	1	1
14,17	12,7	8,753	3	7	1
16,46	8,84	9,369	2	6	1
13,92	8,48	14,651	1	8	1
7,9	9,01	15,038	2	3	1
7,43	13,8	23,527	3	1	1
25,51	11,92	30,245	2	4	1
30,465	14,13	25,389	2	7	1
34,028	8,25	16,891	2	1	1
20,535	10,84	11,958	1	6	1
27,295	17,82	8,753	2	4	1
21,987	23,01	9,369	1	4	1
23,59	32,694	10,061	1	3	1
25,707	18,915	11,135	1	3	1

12,459	16,246	14,651	1	2	1
13,166	11,071	15,038	1	5	1
10,674	5,85	23,527	1	2	2
25,51	24,08	30,245	1	2	2
15,977	12,143	25,389	1	2	1
19,598	13,703	16,891	1	3	2
20,535	7,895	11,958	2	4	1
27,295	8,432	8,753	2	3	2
21,987	32,399	9,369	2	4	2
23,59	21,463	10,061	2	3	3
25,707	25,66	11,135	1	4	3
12,459	10,064	34,028	2	3	2
13,166	14,196	15,038	2	3	1
10,674	5,519	23,527	2	3	2
15,474	6,019	30,245	2	7	3
20,572	14,932	25,389	2	4	2
8,863	10,629	16,891	2	8	1
15,803	10,102	11,958	2	4	2
13,571	4,331	8,753	1	3	3
16,783	12,708	19,369	1	6	2
11,868	6,772	10,061	1	2	1
10,389	4,38	14,651	2	4	2
12,854	9,713	15,038	1	6	1
25,389	4,99	23,527	2	1	2
16,891	6,057	20,245	2	4	2
11,958	13,774	25,389	2	4	3
8,753	7,741	16,891	2	5	3
9,369	4,255	11,958	1	5	2
10,061	22,221	18,753	1	4	1
11,135	11,62	19,369	2	3	2
14,651	12,207	10,061	2	6	2
15,038	6,824	13,8	2	2	2
23,527	14,303	11,92	2	4	1
30,245	8,739	14,13	2	6	1
25,389	8,301	8,25	3	1	1
16,891	6,184	10,84	2	4	1
11,958	8,631	17,82	2	4	1
16,753	13,792	23,01	2	5	1
14,369	6,653	12,694	3	5	2
10,061	8,593	8,915	2	6	2
25,389	7,161	16,246	1	3	2

16,891	8,275	11,071	1	2	3
11,958	6,595	15,85	1	5	3
8,753	9,802	32,399	2	5	3
9,369	9,622	21,463	2	6	2
10,061	7,868	25,66	2	4	1
11,135	14,04	10,064	1	6	1
14,651	14,887	14,196	2	1	1
15,038	8,484	15,519	3	4	2
23,527	6,661	26,019	2	4	2
30,245	7,259	15,932	1	5	2
25,389	8,663	35,629	2	5	2
16,891	6,503	14,102	3	4	1
11,958	13,052	13,732	2	3	2
18,753	15,856	15,038	1	5	2
19,369	8,484	23,527	2	3	2
10,061	16,358	30,245	3	3	2
25,389	6,184	25,389	2	6	1
16,891	14,051	16,891	2	2	2
11,958	6,367	11,958	2	4	1
8,753	14,704	34,753	1	3	2
9,369	15,429	18,915	1	3	2
10,061	5,695	25,389	1	2	3
11,135	6,157	16,891	1	5	3
14,651	13,388	11,958	1	2	2
15,038	5,958	18,753	1	2	1
23,527	6,298	29,369	2	2	2
30,245	7,62	13,061	2	6	3
15,038	4,976	11,135	2	2	2
23,527	4,681	31,651	3	4	2
30,245	13,123	10,061	3	6	1
25,389	6,04	17,82	3	1	2
16,891	7,014	23,01	2	4	2
21,958	10,828	13,8	2	4	2
32,753	6,661	11,428	1	5	2
16,891	4,354	10,127	2	5	1
11,958	7,999	13,56	1	3	1
22,753	13,656	13,35	2	3	2
19,369	14,502	19,622	2	2	2
10,061	13,519	17,868	3	5	2
11,135	7,468	24,04	3	2	2
14,651	15,637	14,887	2	2	2

15,038	7,229	18,484	1	2	3
23,527	15,656	26,661	2	1	3
30,245	15,627	17,259	3	4	2
25,389	6,982	18,663	1	4	2
16,891	5,388	16,503	1	5	1
11,958	9,155	13,052	1	5	2
28,753	15,781	26,157	1	4	1
29,369	7,282	14,598	1	3	2
26,816	10,127	15,958	1	3	2
27,29	7,972	15,958	1	3	3
25,052	15,627	14,239	2	2	3
25,234	7,548	23,874	2	5	2
25,856	15,139	17,562	2	2	2
18,484	15,656	26,04	2	2	2
26,358	13,986	16,298	1	2	2
16,184	13,701	17,62	2	4	2
24,051	16,702	24,976	2	3	2
26,367	13,642	24,681	2	3	1
24,704	5,429	13,123	2	3	1
25,429	17,791	16,04	1	2	1
25,695	7,014	17,014	2	5	2
16,157	15,009	10,828	1	4	1
23,388	14,502	16,661	2	3	2
25,958	14,502	24,354	2	6	2
17,327	10,132	17,999	3	1	2
15,139	10,638	26,751	3	4	1
19,958	10,968	13,656	2	4	1
17,951	7,076	33,972	1	5	1
10,968	8,631	18,142	2	5	2
17,076	11,063	8,795	3	4	2
18,631	18,631	17,349	2	3	1
24,681	8,374	24,502	2	3	1
18,631	17,972	11,063	1	4	2
18,374	8,142	14,704	2	3	1
16,184	18,795	15,337	2	2	2
16,816	17,349	14,819	2	1	3
12,184		18,688	2	2	2
23,695		15,958	1	5	2
23,042		6,324	1	4	1
14,774		24,646	2	3	2
24,887		23,774	2	8	2

15,967		14,502	2	4	2
25,042		10,132	2	3	2
25,306		26,982	2	1	1
17,312		16,289	3	4	2
23,376		17,349	3	4	2
13,656		29,952	2	1	2
14,502		15,637	2		2
23,519		7,229	1		2
17,468		15,656	2		2
13,376		15,627	1		2
15,627		16,982	2		1
14,976		15,388	2		1
24,965		9,155	3		1
25,865		17,29	3		2
17,62		18,51	2		1
23,519		12,207	2		2
17,312		16,824	2		2
25,695		14,303	2		1
18,374		18,739	2		2
18,354		18,301	2		2
17,548		16,184	1		3
17,868		18,631	1		3
15,181		13,792	1		2
13,052		16,653	2		2
15,637		18,593	1		2
10,638		17,161	2		2
15,781		18,275	2		2
17,282		16,595	2		2
10,127		19,802	1		1
17,972		16,702	1		1
15,627		13,642	1		1
17,548		15,429	2		2
25,139		17,791	2		1
15,656		17,014	1		1
13,986		15,009	1		1
13,701		14,502	2		1
18,052		16,595	1		2
24,187		13,641	2		1
15,921		14,04	3		2
24,027		7,951	2		1
16,911		14,819	2		2

15,042		18,282	
16,427		8,992	

%Rap2b (+) vacuoles					
GFP-Rap2b wt	GFP-Rap2b CAAX	GFP-Rap2b wt	GFP-Rap2b CAAX	GFP-Rap2b wt	GFP-Rap2b CAAX
60%	0%	66%	15%	86%	25%
70%	2%	71%	28%	77%	24%
56%	5%	64%	27%	74%	23%
53%	14%	83%	13%	93%	11%
61%	21%	63%	41%	61%	21%
67%	16%	63%	27%	77%	37%
74%	24%	77%	22%	71%	26%
68%	17%	68%	18%	93%	19%
49%	19%	59%	34%	79%	14%
55%	23%	51%	24%	75%	34%

FFU/ml (RU)		
GFP-Control	GFP-Rap2b wt	GFP-Rap2b CAAX
4,170940171	2,87394958	1,212121212
3,675285714	2,608974359	1,016129032
3,97765443	2,560283688	1,155172414
4,2665435	2,360759494	1,183673469
4,918181818	2,452513966	2,70212766
4,377643333	2,762711864	4,29787234
5,86666661	2,503546099	4,16
4,76541034	2,5	5,170212766
4,667754383	2,775193798	4,117647059
5,066037736	2,948275862	4,0625
4,361702128	3,310344828	5,16
5,235294118	2,846846847	4,25
4,185185185	2,677419355	5,246153846
4,205479452	2,482517483	5,984848485
4,166666667	0,45	5,113207547
	2,542635659	4,714285714
4,889976463	3,770833333	5,170731707
5,734693878	2,734513274	4,304347826

4,177654214	1,021505376	
4,779887119	2,263157895	
		5,195121951
	2,911392405	6,559322034
	2,972972973	5,314814815
	2,71	4,291666667

Figure 3

Vacuole diameter (um)		Vacuole diameter (um)	
siRNA Control	siRNA Rap2b	siRNA Control	siRNA EPAC
9.512	8.138	2.582	8.868
7.708	10.189	2.304	3.053
4.285	7.116	1.881	2.620
4.934	8.871	1.316	6.888
8.524	16.915	2.039	5.504
4.717	11.449	3.652	2.343
7.184	7.116	4.888	7.662
8.458	13.444	7.415	6.547
6.638	10.294	1.904	6.124
3.001	11.584	7.415	8.220
6.135	7.720	1.790	7.715
4.738	6.194	8.301	4.813
5.958	9.383	4.233	8.340
5.958	9.689	3.706	3.468
4.239	6.620	1.388	3.216
3.771	9.957	2.153	2.171
7.506	6.426	7.052	2.400
10.127	11.458	1.582	2.264
6.194	8.704	7.117	5.386
7.736	7.116	9.076	1.493
4.875	6.679	5.880	1.125
4.681	9.098	4.773	6.878
3.182	7.398	2.628	2.273
5.842	17.873	2.708	6.756
6.769	5.534	2.058	10.636
2.791	6.194	7.960	4.973
6.711	5.387	4.297	7.223
4.285	5.305	4.009	7.868
7.799	5.118	1.864	3.129

6.751	6.624	8.990	1.585
3.758	6.183	2.648	7.741
4.900	9.098	3.222	3.028
5.037	8.606	5.589	2.572
5.962	4.634	9.056	8.969
4.959	10.560	4.450	6.562
5.534	7.295	2.445	5.437
7.532	5.921	1.921	10.271
3.592	3.854	6.079	6.342
3.777	4.875	6.491	10.000
4.468	8.676	2.637	10.000
3.758	9.581	2.489	8.833
7.493	9.553	2.154	3.034
3.426	5.737	2.573	8.517
5.737	9.281	2.232	6.769
4.860	13.477	3.148	5.505
5.080	6.415	7.415	8.226
5.729	4.262	7.415	7.494
7.650	7.726	8.301	6.547
3.447	7.205	4.233	6.174
7.312	9.388	3.706	8.340
5.118	8.606	1.388	3.494
9.445	7.619	1.582	8.089
6.460	6.426	7.117	7.720
8.138	5.337	9.076	4.852
8.374	7.188	5.880	2.224
8.569	10.189	4.773	2.371
7.238	5.673	2.628	2.286
7.761	5.962	2.058	5.366
5.231	8.871	7.960	1.454
3.159	5.245	4.297	1.093
5.481	4.676	4.009	6.848
10.849	5.032	1.864	2.193
5.826	14.430	8.990	6.831
7.282	5.264	2.648	3.216
10.127	5.133	3.222	8.684
7.972	5.080	4.450	4.973
5.737	5.369	2.423	7.199
7.493	8.862	1.975	7.990
5.245	6.354	6.491	3.195
5.737	5.152	2.616	1.734

3.904	12.980	2.489	7.836
3.653	8.102	2.128	3.896
3.280	7.023	2.552	2.552
3.910	5.208	2.207	8.969
5.921	5.450	3.148	6.562
4.027	6.426	2.577	5.440
6.700	4.860	2.313	8.271
5.042	10.922	1.825	6.342
3.531	5.962	1.288	4.040
3.796	5.569	4.888	7.951
8.177	5.094	1.622	4.819
4.819	9.427	7.868	8.282
8.388	5.921	4.040	8.992
8.871	6.354	4.887	6.427
6.319	12.016	1.484	4.965
5.075	10.822	1.428	5.865
6.003	6.433	1.259	5.347
5.166	5.792	8.663	2.582
7.532	4.875	6.503	5.009
7.312	7.708	1.052	3.732
4.193	7.418	6.157	1.467
6.214	5.534	4.598	6.367
6.023	7.752	5.958	8.188
4.639	6.671	5.958	1.312
4.655	4.753	4.239	7.312
5.152	5.152	1.874	4.303
3.639	8.295	1.562	6.324
7.726	6.722	1.127	6.040
7.506	7.726	6.298	4.739
6.003	7.075	7.620	1.646
7.720	10.265	4.976	5.388
3.639	7.147	4.681	3.774
5.826	11.863	1.123	7.951
5.921	8.191	6.040	7.972
4.549	7.401	1.014	5.627
4.900	9.072	1.828	7.548
5.942	5.652	6.661	5.139
	5.826	4.354	5.656
		1.999	1.986
		6.751	3.701
		1.656	3.052

		4.774	4.187
		4.887	5.921
		5.967	4.027
		5.042	6.911
		5.306	5.042
		1.312	3.641
		3.376	7.620
		3.656	3.519
		4.502	7.312
		3.519	5.695
		1.468	8.374
		3.376	8.354
		5.627	7.548
		4.976	7.868
		4.965	5.181
		5.865	1.052
		5.637	5.781
		1.638	7.282
		1.127	

FFU/ml (RU)			
siRNA Control	siRNA Rap2b	siRNA Control	siRNA EPAC
4,409252669	5,308868502	4,290322581	5,644808743
3,4	5,473498233	4,34516129	4,886524823
4,469594595	6,3374613	4,324099723	4,960264901
3,438485804	5,358552632	3,374655647	6,126760563
4,382262997	5,686131387	4,471471471	5,127659574
4,354570637	5,559027778	4,376731302	4,940298507
4,353896104	6,335443038	3,362776025	6,990291262
5,438311688	6,54516129	4,671717172	6,508474576
4,381756757	5,393846154	4,082568807	4,893939394
3,415335463	5,471631206	4,304469274	5,971014493

Figure 4

%EPAC (+) vacuoles		%Rap2b (+) vacuoles	
C. burnetii wt	*C. burnetii* Tn292	*C. burnetii* wt	*C. burnetii* Tn292
40%	0%	46%	5%
50%	2%	51%	8%

46%	5%	44%	7%
43%	4%	43%	3%
51%	11%	53%	4%
47%	6%	43%	7%
43%	4%	47%	3%
35%	7%	48%	10%
41%	9%	39%	3%
35%	3%	31%	5%

Figure 5

% Colocalización *C.burnetii*/mCherry Cox			% Colocalización *C.burnetii*/Dextran		
GFP-Control	GFP-Rap2b wt	GFP-Rap2b CAAX	GFP-Control	GFP-Rap2b wt	GFP-Rap2b CAAX
53,02%	14,63%	60,85%	62,21%	33,13%	63,38%
67,49%	16,44%	50,87%	58,86%	28,86%	68,82%
57,90%	19,90%	66,33%	59,24%	24,18%	51,54%
50,62%	14,54%	49,80%	54,69%	20,66%	50,45%
49,00%	23,16%	52,30%	51,52%	21,52%	50,19%
55,80%	24,69%	60,85%	69,22%	27,25%	60,77%
41,17%	14,43%	50,87%	73,88%	26,65%	62,74%
58,05%	18,65%	66,33%	78,24%	23,17%	73,76%
60,49%	31,75%	49,80%	79,53%	34,45%	73,68%
69,06%	13,75%	52,30%	65,81%	24,19%	57,86%
			69,25%	20,64%	61,56%
			66,99%		52,47%

Figure 6

Vacuole diameter (um)			CRV number/Cell		
GFP-Control	GFP-Rap2b wt	GFP-Rap2b CAAX	GFP-Control	GFP-Rap2b wt	GFP-Rap2b CAAX
11.443	13.996	7.496	2	3	2
7.215	11.753	6.183	2	4	1
5.530	14.855	15.147	1	3	1
8.093	13.310	14.592	2	2	1
14.717	13.531	5.152	2	1	1
6.711	13.121	6.978	3	2	2
14.885	14.245	7.130	2	2	2
6.426	10.003	14.934	1	4	1
4.691	10.798	15.556	2	3	2
3.809	11.053	6.844	3	2	1

2.207	15.037	6.003	2	1	2
3.310	13.996	7.188	1	4	2
7.944	15.888	6.653	1	4	3
10.974	14.860	5.673	1	1	3
7.554	10.208	14.435	2	4	2
7.075	10.433	9.302	2	4	1
6.460	15.830	5.888	2	4	2
15.133	10.792	7.836	2	3	3
15.826	11.450	15.333	2	1	2
7.676	15.938	15.737	1	3	1
9.499	11.792	5.208	1	3	2
7.959	15.133	10.317	1	3	3
6.897	13.751	13.242	1	3	2
8.617	16.873	6.516	1	3	1
7.532	10.532	7.622	1	1	2
5.296	16.003	10.210	1	4	3
7.305	10.059	8.096	1	7	2
5.333	8.165	14.285	1	1	2
6.613	10.403	13.447	1	2	2
7.335	10.133	4.691	1	4	1
6.908	8.796	6.550	1	4	2
4.764	8.138	14.676	1	3	1
18.595	10.189	15.737	1	3	1
15.305	7.116	9.634	1	2	1
16.679	8.871	14.463	1	2	1
15.075	16.915	15.534	2	2	1
15.556	11.449	6.214	2	2	1
16.653	7.116	7.345	1	2	1
15.754	13.444	13.317	2	3	1
17.013	10.294	10.103	1	4	1
16.433	11.584	17.365	2	3	2
15.974	7.720	10.962	2	4	2
13.860	6.194	15.170	3	3	2
14.840	9.383	10.736	3	4	2
14.164	9.689	10.788	2	3	1
14.463	6.620	10.638	1	3	2
16.183	9.957	10.617	2	3	2
14.959	6.426	10.096	3	2	2
15.032	11.458	16.433	2	4	2
10.827	8.704	17.092	1	3	2
14.860	7.116	10.467	2	4	2

14.245	6.679	19.178	3	3	2
14.369	9.098	14.978	2	6	1
15.521	7.398	14.245	1	2	1
14.934	17.873	19.553	2	4	1
15.118	5.534	10.698	1	2	2
14.216	6.194	16.460	2	1	1
13.426	5.387	10.168	2	4	2
15.673	5.305	15.517	3	4	2
15.673	5.118	14.369	2	2	2
16.490	6.624	15.805	1	2	2
17.009	6.183	10.305	1	4	1
15.656	9.098	15.003	2	3	2
16.894	8.606	10.708	2	3	2
15.938	4.634	9.512	2	2	2
14.840	10.560	7.708	2	4	1
10.354	7.295	14.285	2	2	1
16.369	5.921	14.934	3	1	1
16.553	3.854	8.524	2	4	1
15.876	4.875	5.118	2	4	1
16.147	8.676	7.184	2	2	1
10.307	9.581	8.458	3	2	2
10.506	9.553	6.638	2	2	2
8.446	5.737	13.001	1	3	2
9.512	9.281	6.135	1	2	3
7.708	13.477	14.738	1	2	3
14.285	6.415	15.958	2	2	3
14.934	4.262	5.958	2	2	2
8.524	7.726	14.239	2	4	1
11.174	7.205	13.771	1	6	1
7.184	9.388	7.506	2	1	1
8.458	8.606	7.312	3	4	2
6.638	7.619	6.194	2	4	2
13.001	6.426	7.736	1	2	2
6.135	5.337	14.875	2	2	2
14.738	7.188	14.681	3	4	1
15.958	10.189	13.182	2	3	2
15.958	5.673	5.842	1	5	2
14.239	5.962	6.769	2	3	2
13.771	8.871	12.791	3	3	2
7.506	5.245	6.711	2	3	1
10.127	4.676	14.285	2	2	2

6.194	5.032	7.799	2	4	1
7.736	14.430	6.751	1	3	2
14.875	5.264	13.758	1	3	2
14.681	5.133	14.900	1	2	3
6.319	5.080	15.037	1	5	3
15.075	5.369	15.962	1	2	2
6.003	8.862	14.959	1	2	1
5.166	6.354	15.534	2	2	2
9.445	5.152	7.532	2	2	3
6.460	12.980	13.592	2	2	2
8.138	8.102	13.777	3	4	2
7.532	7.023	14.468	3	6	1
7.312	5.208	13.758	3	1	2
4.193	5.450	7.493	2	4	2
16.214	6.426	13.426	2	4	2
6.023	4.860	15.737	1	2	2
14.639	10.922	14.860	2	2	1
14.655	5.962	15.080	1	2	1
5.152	5.569	15.729	2	4	2
13.639	5.094	7.650	2	2	2
7.726	9.427	13.447	3	1	2
7.506	5.921	15.695	3	4	2
6.003	6.354	8.374	2	4	2
7.720	12.016	8.569	1	2	3
13.639	10.822	7.238	2	2	3
15.826	6.433	7.761	3	4	2
15.921	5.792	15.231	1	3	2
4.549	4.875	13.159	1	2	1
14.900	7.708	15.826	1	2	2
15.942	7.418	7.282	1	3	1
14.717	5.534	15.481	1	4	2
13.777	7.752	7.972	1	2	2
13.796	6.671	15.737	1	1	3
14.676	4.753	7.493	2	4	3
15.556	5.152	15.245	2	4	2
15.118	8.295	15.737	2	5	2
15.042	6.722	13.904	2	5	2
13.258	7.726	13.653	1	4	2
15.032	7.075	13.280	2	3	2
14.934	10.265	13.910	2	3	2
16.403	7.147	15.921	2	3	1

14.691	11.863	14.027	2	3	1
15.305	8.191	6.700	1	2	1
15.080	7.401	15.042	2	5	2
15.032	9.072	13.531	1	4	1
8.738	5.652	13.796	2	3	2
15.194	5.826	8.177	2	2	2
5.942	5.974	14.819	3	1	2
6.311	6.635	8.388	3	4	1
15.830	5.938	8.524	2	4	1
6.183	6.894	15.929	1	5	1
15.587	4.717	16.911	2	5	2
15.569		7.542	3	4	2
9.478		8.936	2	3	1
8.093		14.845	2	3	1
14.885		14.753	1	4	2
16.433		17.956	2	3	1
17.205			2	2	2
15.231			2	1	3
			2	2	2
			1	5	2
			1	4	1
			1	3	2
			1	3	2
			1	4	2
			1	3	2
			1	1	1
			1	4	2
			1	4	2
			2	1	2
			2	4	2
			1	3	2
			2	2	2
			1	1	2
			2	4	1
			2	4	1
			3	2	1
			3	2	2
			1	4	1
			2	3	2
			1	3	2
			2	4	1

1	3	2
2	2	2
1	2	3
1	2	3
1	4	2
2	2	2
1	1	2
2	4	2
2	4	2
2	2	2
1	2	1
1	4	1
1	3	1
2	5	2
1	5	1
1	3	1
1	3	1
1	2	1
1	5	2
1	2	1
1	2	2
1	2	1
1	1	
1	4	
1	4	
1	2	
1	2	
1	4	
1	3	
1	3	
1	3	
1	2	
1	2	
	2	

% LC3 (+) Vacuoles		
GFP-Control	GFP-Rap2b wt	GFP-Rap2b CAAX
13,30%	27,51%	12,55%
14,33%	28,82%	14,20%

13,30%	26,68%	17,09%
16,42%	28,05%	13,84%
17,91%	20,19%	14,62%
12,38%	27,88%	22,40%
13,16%	26,20%	17,40%
14,17%	27,11%	23,26%
13,12%	27,24%	14,78%
	24,42%	16,90%
	27,16%	24,73%
	22,85%	
